# Semen Ziziphi Spinosae attenuates blood–brain barrier dysfunction induced by lipopolysaccharide by targeting the FAK-DOCK180-Rac1-WAVE2-Arp3 signaling pathway

**DOI:** 10.1038/s41538-022-00142-6

**Published:** 2022-06-02

**Authors:** Huayan Liu, Xin Zhang, Yujiao Liu, Nian Xin, Yulin Deng, Yujuan Li

**Affiliations:** 1 School of Life Science, Beijing Institute of Technology, 100081 Beijing, China; 2 BIT&GS Technologies Co. Ltd, 100074 Beijing, China

**Keywords:** Quality of life, Blood-brain barrier

## Abstract

Semen Ziziphi Spinosae (SZS) has been extensively used in the daily diet as a functional food for neuroprotective health-benefit in China for many years. However, the neuroprotective mechanism of SZS associated with blood–brain barrier (BBB) integrity remains unexplored. The present study suggests SZS could protect against lipopolysaccharide (LPS)-induced BBB dysfunction. Proteomics indicate that 135 proteins in rat brain are significantly altered by SZS. These differentially expressed proteins are mainly clustered into cell–cell adhesion and adherens junctions, which are closely related with BBB integrity. SZS reversed LPS-induces BBB breakdown by activating the FAK-DOCK180-Rac1-WAVE2-Arp3 pathway. Molecular docking between signaling pathway proteins and identified SZS components in rat plasma reveals that 6”‘-feruloylspinosin, spinosin, and swertisin strongly binds to signaling proteins at multiple amino acid sites. These novel findings suggest a health benefit of SZS in prevention of cerebral diseases and contributes to the further application of SZS as a functional food.

## Introduction

The blood–brain barrier (BBB) is formed primarily with cerebral microvessel endothelial cells (CMECs). It is also covered with basement membranes and surrounded by pericytes and astrocytes^1^. CMECs are connected by tight junctions (TJs) and adherens junctions (AJs) which control the permeability of the BBB^2^. BBB plays an important role in maintaining the normal physiological function of the central nervous system (CNS), by preventing neurotoxic plasma components and pathogens from entering the brain^3^. Studies have shown that the early BBB breakdown contributes to the process of central nervous diseases such as Alzheimer’s disease, Parkinson’s disease, Huntington’s disease, depression and so on^4^. Therefore, it is considered that regular use of functional food beneficial to BBB integrity has protective effects on aging and many neurological diseases.

Semen ziziphi spinosae (SZS, named Suanzaoren in Chinese) is the dried ripe seeds of *Ziziphus jujuba* Mill. var. *spinosa* (Bunge) Huex H. F. Chou. SZS is one homology plant used as both functional food and herbal medicine^5^. As a functional food, the consumption of SZS in the daily diet has increased dramatically in China because of its nutritive values and medicinal effects. SZS is widely made into dietary products, including sleeping tea, beverages, jellies, and yogurt^6^. SZS has also been added directly to foods, such as porridge, by the locals. The extensive use of SZS suggests its potential as a functional food for preventing the onset of neurological disorders. As a traditional Chinese medicine, it has been used for sedation and hypnosis for thousands of years^7^. SZS also exhibits anti-depressant, antioxidant, and anxiolytic effects^8,9^. SZS and its active components, such as spinosin (SPI), may also regulate immune function, improve learning and memory, and prevent Alzheimer’s disease^10^. It is known that the excessive production of reactive oxygen species and inflammatory factors could promote the degradation of junction proteins, and then result in BBB destruction. It has been reported that SZS exhibits significant antioxidant and anti-inflammatory effects, which might suggest SZS could protect the BBB by inhibiting oxidation and inflammatory response, and then potentially prevent central nervous diseases. However, up to know, research on the effects of SZS on BBB seems unavailable.

In recent years, mass spectrometry (MS)-based proteomics has become a necessary tool to understand biological processes at the protein level, identify diagnostic and prognostic markers, and elucidate the underlying mechanisms of disease. MS also provides insight into the prevention and treatment of health disorders^11^. Lipopolysaccharide (LPS) is widely utilized to develop in vivo and in vitro BBB damage models because it induces neuroinflammation and oxidative stress^2,12^. Oxidative stress or neuroinflammation could damage BBB integrity by degrading tight junction proteins between the CMECs, promoting paracellular leakage, or modulating the Nrf2 antioxidant and NF-κB inflammatory pathways^13^. Alterations in cellular matrix adhesion also contribute to increased BBB permeability resulting from brain injury^14^. Thus far, the global proteomic profiling of the rat brain following treatment with SZS has not been established. Furthermore, the underlying mechanism of SZS as a functional food against BBB breakdown following LPS exposure has not been revealed.

In the present study, the protective effect of SZS on LPS-induced BBB damage was examined through histomorphology, permeability, biochemical assay, and expression of junction proteins in rat brain and human cerebral microvascular endothelial cells (hCMEC/D3). The global proteomic profile and possible underlying mechanism of SZS against BBB damage were carried out using nano liquid chromatography (LC)-mass spectrometer (MS) analysis and western blot validation. The chemical composition in SZS decoction and in the plasma from rats orally administered with SZS was identified. The interaction between in vivo SZS components and signaling pathway proteins was evaluated in molecular docking experiments. The results may contribute to the further use of SZS for health benefits and shed light on the prevention and treatment of neurological diseases.

## Results

### SZS attenuates rat brain and cell injury induced by LPS

**Fig. 1:**
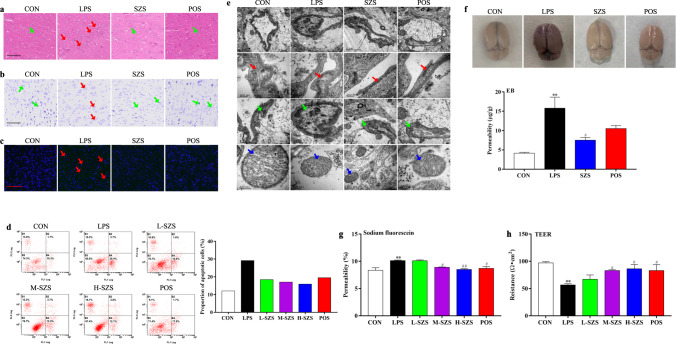
SZS attenuates rat brain injury induced by LPS. H&E (**a**, inflammatory and normal cells are marked with red and green arrows, respectively; the length of scale bar was 50 μm), Nissl (**b**, Nissl bodies are marked with red arrows; the length of scale bar was 50 μm) and TUNEL (**c**, green fluorescence for apoptosis and marked with red arrows; the length of scale bar was 200 μm) staining of rat cortex in each group. Apoptosis evaluation in hCMEC/D3 cells by flow cytometry (**d**). FL1 represents FITC fluorescence and FL3 represents PI fluorescence. Ultra-structure of rat brain observed by TEM (**e**, intercellular space, pericyte, and mitochondrion are marked with red, green, and blue arrows; the length of scale bars were 2 μm, 200 nm, 1 μm and 200 nm, respectively). Photos of rat brain after EB injection and EB content in rat cortex of each group (**f**). Sodium fluorescein leakage (**g**) and TEER value (**h**) in hCMECs/D3 cells from each group. Data are expressed as mean ± SD (*n* = 9). The doses of L-SZS, M-SZS, and H-SZS were 50, 100, and 200 μg/mL, respectively. Compared with the CON group, *P < 0.05 and **P < 0.01. Compared with the LPS group, ^#^*P* < 0.05, ^##^*P* < 0.01.

The results of hematoxylin and eosin (H&E) staining were shown in Fig. 1a. Compared with the control (CON) group, LPS exposure resulted in a significant inflammatory infiltration (red arrows), nuclei shrinkage (green arrows), and cell swelling in the rat cortex. SZS inhibited this damage and restored cell morphology. Vc showed a similar protective effect to that of SZS. Nissl and terminal deoxynucleotidyl transferase-deoxyuridine triphosphate nick end-labeling (TUNEL) staining indicated that LPS treatment resulted in decreased Nissl bodies (Fig. 1b, red arrows) and increased apoptotic cells (green fluorescence marked in red arrows in Fig. 1c), which was dramatically improved by SZS administration to LPS-exposed rats. Flow cytometry analysis revealed that SZS (50, 100 and 200 µg/mL) significantly inhibited cell apoptosis induced by LPS (Fig. 1d). The proportion of apoptotic cells (B4 + B2) decreased from 29.1% in LPS group to 18.4%, 17% and 15.9% in L-SZS, M- SZS and H-SZS groups respectively, showing a dose-dependent decreasing trend. Ultrastructure analysis by transmission electron microscopy (TEM) (Fig. 1e) indicated that LPS exposure induced a larger intercellular space (red arrows), swollen astrocytes (green arrows), and damaged mitochondrial structures (blue arrows). In the SZS group, the damaged ultrastructure was repaired, including a narrower cell space, suppressed swelling of astrocytes, and an intact mitochondrial inner membrane and clear cristae structures. Taken together, these results indicate that SZS protects damaged tissue morphology and ultrastructure, and inhibits apoptosis and necrosis induced by LPS.

### SZS decreases permeability in rat and hCMEC/D3 cells following LPS exposure

Evans blue (EB) extravasation in rat cortex (Fig. 1f) suggested that EB levels in the LPS group were remarkably higher compared with that in the CON group (*P* < 0.01). SZS and Vc treatment dramatically reduced EB content by 52.5% and 33.5%, respectively, compared with the LPS group. In LPS-treated hCMEC/D3 cells, sodium fluorescein penetration was high and trans-endothelial electrical resistance (TEER) values were low (Fig. 1g, h). Following treatment with SZS, the low-dose group (50 µg/mL) exhibited no significant effect on permeability and TEER values. Medium (100 µg/mL) and Vc considerably reduced sodium fluorescein penetration and increased TEER values (*P* < 0.05). High-dose (200 µg/mL) SZS could reduce the permeability of sodium fluorescein by 16.0% (*P* < 0.01) and increased TEER values by 53.5% (*P* < 0.05). Based on these results, we concluded that SZS reduced BBB permeability in rats and protected BBB integrity in a cell model of LPS exposure.

### SZS decreases oxidative stress and inflammatory levels in LPS-exposed rat and hCMEC/D3 cells

**Fig. 2:**
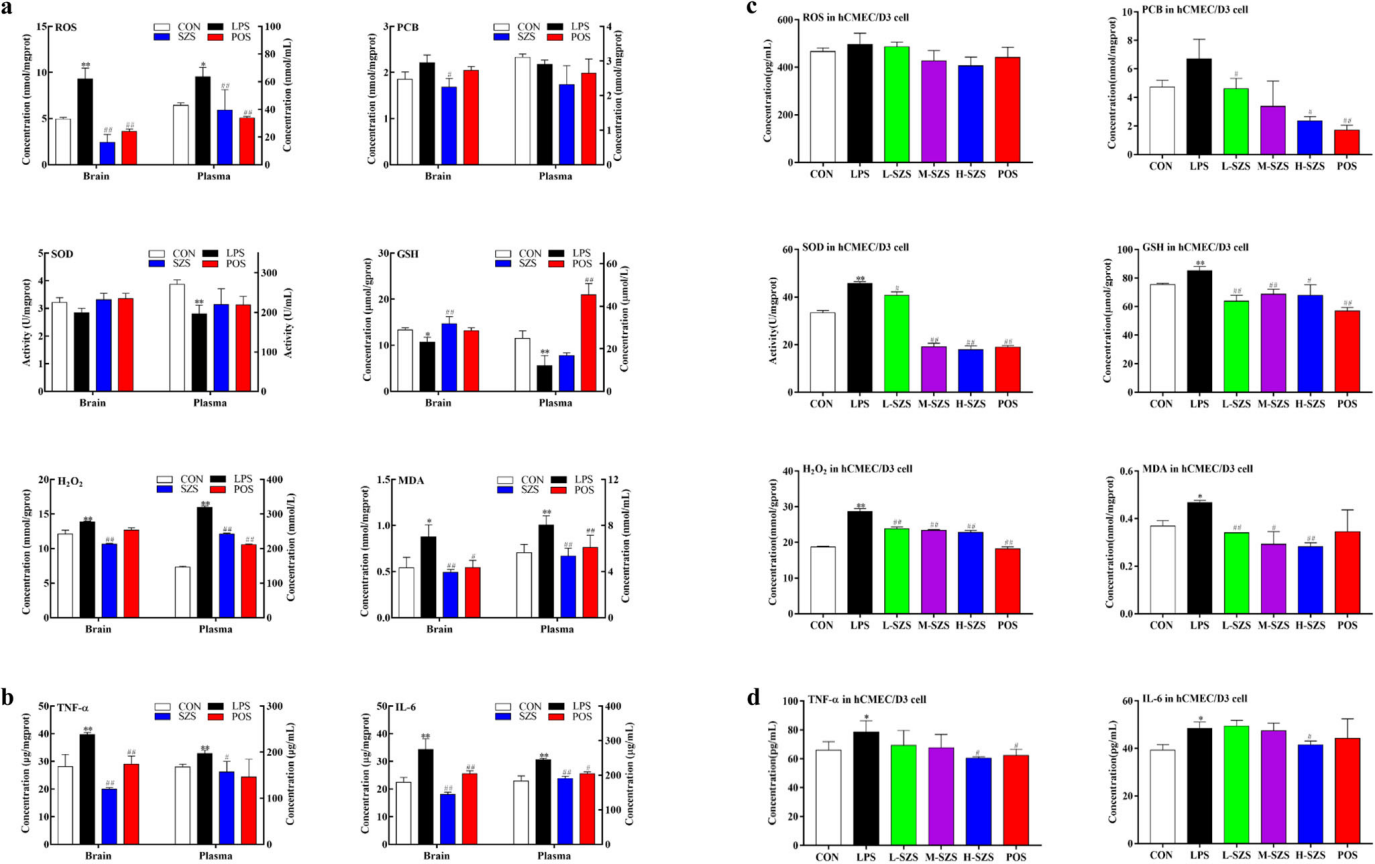
SZS improves oxidative stress and inflammation levels in rats exposed to LPS. Levels of oxidative stress and inflammatory factors in rat brain, plasma (**a** and **b**), and hCMEC/D3 cells (**c** and **d**) determined by corresponding assay kits. Data are expressed as the mean ± SD (*n* = 9). The doses of L-SZS, M-SZS, and H-SZS were 50, 100, and 200 μg/mL, respectively. Compared with the CON group, *P < 0.05 and **P < 0.01. Compared with the LPS group, ^#^*P* < 0.05, ^##^*P* < 0.01.

The effects of SZS on the antioxidant capacity of rat cortex and plasma were evaluated by superoxide dismutase (SOD) activity and the levels of glutathione (GSH), malondialdehyde (MDA), hydrogen peroxide (H_2_O_2_), reactive oxygen species (ROS), and protein carbonyls (PCB). GSH levels and SOD activity in rat plasma were significantly reduced by LPS compared with the CON group, whereas SZS tended to reverse these effects without significant differences (Fig. 2a, b). There was no significant difference in SOD activity in rat brains from the LPS and SZS groups. SZS remarkably increased GSH level in rat brain compared with LPS group. The increased ROS, MDA, and H_2_O_2_ levels in rat brains and plasma were abrogated by SZS treatment. No significant differences were observed in the PCB content of rat brain and plasma between the CON and LPS groups, whereas SZS administration markedly decreased PCB levels in rat brain compared with the LPS group. In summary, SZS inhibited oxidative stress levels in rat plasma and cortex following LPS exposure, potentially accounting for the protective effects against BBB damage. Using TEM, we observed that SZS improved mitochondrial structures in the rat cortex, which may have increased the antioxidant effects. Compared with the CON group, the levels of IL-6 and TNF-α in rat cortex and plasma in the LPS group were significantly elevated (*P* < 0.01, Fig. 2b). SZS administration reduced inflammatory factor levels in the rat cortex and plasma. These results were confirmed by assessing the inflammatory infiltrates following SZS treatment by H&E staining.

In hCMEC/D3 cells exposed to LPS, markedly increased inflammatory and oxidative stress levels were observed (Fig. 2c, d). When hCMEC/D3cells were treated with 50, 100, and 200 μg/mL of SZS, inflammatory and oxidative injury were ameliorated, accompanied by decreased levels of IL-6, TNF-α, GSH, MDA, H_2_O_2_, and ROS in a dose-dependent manner. Collectively, SZS decreased oxidative stress and inflammatory levels in LPS-exposed rat and hCMEC/D3 cells.

### SZS protected BBB integrity by increasing the expression of TJ and AJ proteins

**Fig. 3:**
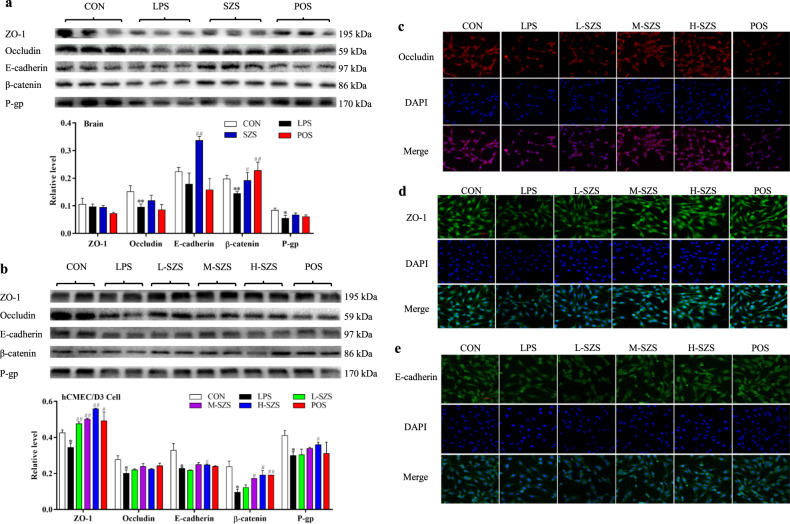
SZS increases the expression of junction proteins, P-gp. The expression of ZO-1, occludin, E-cadherin, β-catenin, P-gp, and the ratio of F/G-actin in rat brain (**a**) and hCMEC/D3 cells (**b**) was determined by western blot analysis. Immunofluorescence (IF) staining was used to examine the expression and distribution of occludin (**c**), ZO-1 (**d**), and E-cadherin (**e**) in hCMEC/D3 cells for all groups (the length of scale bars were 50 μm). Data for the western blot were normalized with total protein levels as an internal reference, and the total protein gels are shown in Supplementary Figure 1. All blots or gels derive from the same experiment and that they were processed in parallel. Data are expressed as the mean ± SD (*n* = 9). The doses of L-SZS, M-SZS, and H-SZS were 50, 100, and 200 μg/mL, respectively. Compared with the CON group, *P < 0.05 and **P < 0.01. Compared with the LPS group, ^#^*P* < 0.05, ^##^*P* < 0.01.

Expression levels of tight junction proteins (TJ, zona occludens 1 (ZO-1) and occludin), adherens junction proteins (AJ, epithelial cadherin (E-cadherin) and β-catenin), and P-glycoprotein (P-gp, a transporter involving in BBB integrity regulation) are shown in Fig. 3a. The relative levels of the proteins were expressed as the ratio of the gray value of the target band to that of the total proteins in the same sample^15^. The gel images for total protein are shown in Supplementary Fig. S1. Occludin, β-catenin, and P-gp expression in the rat cortex following LPS exposure was dramatically decreased (*P* < 0.05). SZS elevated the expression of β-catenin (*P* < 0.05), occluding, and P-gp (without significance) compared with the LPS group. No significant change in ZO-1 expression was observed in rats exposed to LPS or SZS. Vc as the positive control upregulated β-catenin expression. SZS increased the expression of junction proteins and P-gp, which may be useful for improving junction structures and maintain BBB integrity.

Expression of all TJ and AJ proteins and P-gp was significantly decreased in LPS-treated hCMEC/D3cells (*P* < 0.05, Fig. 3b) compared with the CON group. SZS (50, 100, and 200 μg/mL) treatment significantly upregulated the expression of ZO-1 (*P* < 0.01) and β-catenin in a dose-dependent manner. SZS at the highest dose (200 μg/mL) increased the expression of E-cadherin and P-gp by 9.07% and 19.9% (*P* < 0.05), respectively. Three doses of SZS and Vc slightly increased occludin expression. An immunofluorescence (IF) assay indicated that the three doses of SZS increased occludin (Fig. 3c), ZO-1 (Fig. 3d), and E-cadherin (Fig. 3e) expression in a dose-dependent manner compared with LPS-treated hCMEC/D3 cells. SZS improved the distribution of occludin and ZO-1, whereas no significant influence on E-cadherin was evident. Collectively, SZS protects BBB integrity by increasing the expression of junction proteins and P-gp.

### SZS significantly regulated rat cortex proteins based on proteomics

**Fig. 4:**
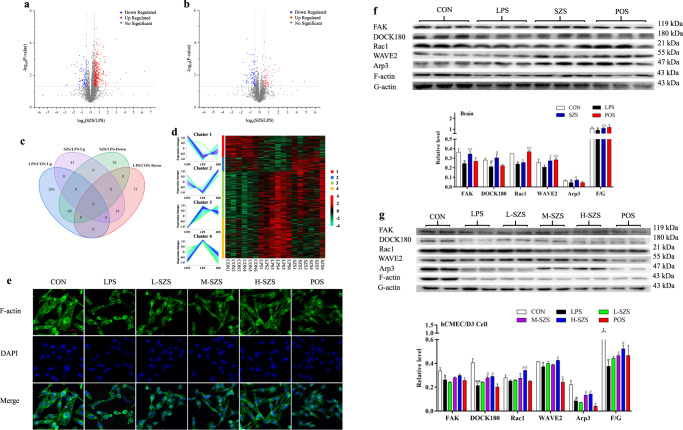
SZS alters cortex proteins in LPS-exposed rats based on proteomics and regulates the FAK-DOCK180-Rac1-WAVE2-Arp3 signaling pathway. Differentially expressed proteins (DEPs) are shown in volcano maps for the LPS/CON group (**a**), SZS/LPS group (**b**), and the Venn map (**c**). All DEPs were classified into four clusters and a heatmap (**d**). Immunofluorescence (IF) staining was used to examine the expression and distribution of F-actin (**e**, the length of scale bars were 50 μm). The expression of FAK, DOCK180, Rac1, WAVE2, and Arp3 in rat cortex (**f**) and hCMEC/D3 cells (**g**) were determined by western blot analysis. Western blot data were normalized with total protein levels as the internal reference, and the total protein gel is shown in Supplementary Figure 2. All blots or gels derive from the same experiment and that they were processed in parallel. Data are expressed as the mean ± SD (*n* = 9). The doses of L-SZS, M-SZS, and H-SZS were 50, 100, and 200 μg/mL, respectively. Compared with the CON group, *P < 0.05 and **P < 0.01. Compared with the LPS group, ^#^*P* < 0.05, ^##^*P* < 0.01.

A total of 3998 proteins were identified based on a label-free proteomic strategy, which is shown by volcano plots (Fig. 4a, b). Differentially expressed proteins (DEPs) (*n* = 387) in the LPS and CON comparison groups (marked as LPS/CON) and DEPs (*n* = 135) in the SZS and LPS comparison groups (marked as SZS/LPS) are depicted in a Venn diagram (Fig. 4c). All DEPs are listed in Supplementary Table S1. In Venn diagram, SZS significantly increased the expression of 14 downregulated proteins induced by LPS and decreased the expression of 40 upregulated proteins induced by LPS. The DEPs in the LPS/CON and SZS/LPS groups were, respectively, categorized into three and two annotation clusters (shown in Table 1) by the DAVID functional tool. The DEPs in the LPS/CON group are primarily involved in cell–cell adhesion, GDP and GTP binding, and intermediate filament and neurofilament cytoskeleton organization. SZS treatment significantly attenuated proteins associated with the dendritic shaft, postsynaptic density, cell junction, and postsynaptic membrane. The expression of cytochrome P450 2D1 (CYP2D1) in the SZS group, validated by western blot analysis, demonstrated a pattern consistent with the MS analysis (shown in Supplementary Fig. S2).

**Table 1:** Annotation clusters of differently expressed proteins (DEPs).

LPS/CON comparison group			
Category	Term	Count	*P* value
Annotation cluster 1 enrichment score: 3.4			
GOTERM_MF	Cadherin binding involved in cell–cell adhesion	15	0.0002
GOTERM_CC	Cell–cell adherens junction	16	0.0002
GOTERM_BP	Cell–cell adhesion	13	0.0013
Annotation cluster 2 enrichment score: 2.84			
GOTERM_MF	GDP binding	8	0.0002
GOTERM_BP	Small GTPase mediated signal transduction	14	0.0010
GOTERM_MF	GTP binding	16	0.0130
Annotation cluster 3 enrichment score: 2			
GOTERM_BP	Intermediate filament cytoskeleton organization	4	0.0038
GOTERM_CC	Neurofilament	3	0.0160
GOTERM_BP	Neurofilament cytoskeleton organization	3	0.0170
SZS/LPS comparison group			
Category	Term	Count	*P* value
Annotation cluster 1 enrichment score: 1.76			
GOTERM_CC	Dendritic shaft	4	0.0093
GOTERM_CC	Dendritic spine	5	0.0220
GOTERM_CC	Postsynaptic density	6	0.0280
Annotation Cluster 2 Enrichment Score: 1.66			
GOTERM_CC	Postsynaptic membrane	6	0.0140
GOTERM_CC	Cell junction	8	0.0280
GOTERM_CC	Postsynaptic density	6	0.0280

To visualize the protein expression patterns in the two comparison groups, DEPs were divided into four clusters (Fig. 4d) using the Mfuzz package (www.bioconductor.org). Compared with the CON group, DEPs in Cluster 1 and 2 were downregulated by LPS exposure, whereas they were restored to normal levels by SZS treatment. Similarly, SZS decreases the expression of DEPs upregulated with LPS in Cluster 3 and 4. The DAVID functional annotation analysis indicated that proteins in Cluster 1 were primarily associated with cadherin binding involved in cell–cell adhesion. Proteins in Cluster 2 and 4 were involved in cell junction and ion transmembrane transport. Proteins in Cluster 3 were mainly associated with the molecular function of GTPase activator activity and Rab GTPase binding.

The proteomics results showed that LPS treatment affects cell–cell adhesion and intermediate filament and neurofilament cytoskeleton organization in rat brains, whereas SZS treatment significantly regulated dendritic spine, postsynaptic density, and cell junction proteins. Cell–cell adhesion includes focal adhesions (FAs) and adherens junction proteins (Ajs). The cell junction consists of tight junctions (TJs) and AJs. Cell–cell adhesion and junction maintain the integrity of the BBB through a series of proteins which include focal adhesion kinase (FAK), E-cadherin, β-catenin, ZO-1, and occludin. The FAs, AJs, and TJs are linked to the actin cytoskeleton through various adaptor proteins. The actin cytoskeleton plays an important role in regulating the stability of endothelial cell contacts and BBB permeability^16^. Disrupted endothelial cell adhesions and junctions may cause reorganization of the cytoskeleton, leading to a breakdown of BBB integrity. Based on our proteomic results regarding AJ and TJ protein determination and F-actin immunofluorescence, it is possible that SZS maintains BBB integrity by regulating cell junctions and the actin cytoskeleton. It has been reported that cytoskeletal reorganization may be regulated by several signaling pathways, including the Ras-related C3 botulinum toxin substrate 1 (Rac1)- Wiskott-Aldrich syndrome protein family member 2 (WAVE2)- actin related protein 2/3 (Arp2/3) pathway^17^. F-actin assembly and small actin driven protrusions, controlled by the Arp2/3 complex, contribute to the regulation of the paracellular permeability of the BBB^18^. It has also been documented that upstream FAK and dedicator of cytokinesis 1 (DOCK180) regulate Rac1^19^. Currently, there are no reports describing LPS-induced BBB damage through the FAK-DOCK180-Rac1-WAVE2-Arp3 signaling pathway. Also, the mechanism of SZS in protecting BBB has not been elucidated. Therefore, whether SZS prevents BBB disruption induced by LPS through the FAK-DOCK180-Rac1-WAVE2-Arp3 signaling pathway was further examined.

### SZS protects BBB integrity via the FAK-DOCK180-Rac1-WAVE2-Arp3 signaling pathway

F-actin expression and distribution in hCMEC/D3 cells in response to LPS exposure, SZS and Vc administration were examined by Immunofluorescence (IF) staining (as shown in Fig. 4e). LPS exposure decreased the expression and distribution of F-actin compared to CON group and SZS administration increased those in a dose-dependent manner. The expression of FAK, DOCK180, Rac1, WAVE2, Arp3 in the rat cortex and hCMEC/D3 cells determined by western blot analysis is presented in Fig. 4f, g, respectively. The relative levels of the proteins were expressed as the ratio of the gray value of the target band to that of total proteins in the same sample. The gels representing total protein are shown in Supplementary Fig. S3. Expression of FAK, DOCK180, Rac1, and the ratio of F-actin to G-actin (F/G) in the rat cortex were markedly decreased following LPS exposure (*P* < 0.05). SZS treatment significantly upregulated the expression of FAK (41.2%, *P* < 0.01), DOCK180 (44.4%, *P* < 0.05), Rac1 (6.7%, *P* < 0.01), WAVE2 (32.5%, *P* < 0.01), and Arp3 (64%, *P* < 0.05), compared with the LPS group. SZS restored the decreased F/G-actin ratio (*P* < 0.01) induced by LPS.

After LPS treatment, the expression of FAK, DOCK180, WAVE2, Arp3, and the F/G ratio in hCMEC/D3 cells were dramatically decreased by 22.1%, 47.3%, 10.5%, 61.4%, and 66.3%, respectively (*P* < 0.05). SZS treatment significantly increased the expression of DOCK180, Rac1, Arp3, and the F/G ratio in a dose-dependent manner. A high dose of SZS (200 μg/mL) increased WAVE2 expression, and three doses of SZS increased FAK expression. In summary, SZS potentially protected BBB integrity in LPS-exposed rats and hCMEC/D3 cells through the FAK-DOCK180-Rac1-WAVE2-Arp3 signaling pathway.

### Identification of SZS components in SZS decoction and rat plasma

Components in SZS decoction and SZS-treated rat plasma were identified by high-resolution signal derivation and compared with reference standards and the literature. According to the measured accurate relative molecular mass, the data analysis processing software was used to calculate the possible error within the specified error range (error within ±5 ppm). By comparing retention times, high-resolution mass data for SZS, blank, and rat plasma, 33 constituents in the SZS decoction and 13 in vivo compounds in rat plasma were identified. For prototype compounds, four were identified unambiguously by comparison with the references, SPI, swertisin (SWE), 6”‘-feruloylspinosin (FSP), and coclaurine (COC). The data for all the identified components in the SZS decoction and rat plasm are listed in Supplementary Tables S2 and S3. In vivo prototype compounds, SPI, SWE, FSP, COC, zizyphusine (ZIZ), and saponarin (SAP) were selected for molecular docking analysis.

### Molecular docking between in vivo SZS components and proteins related to BBB damage

**Fig. 5:**
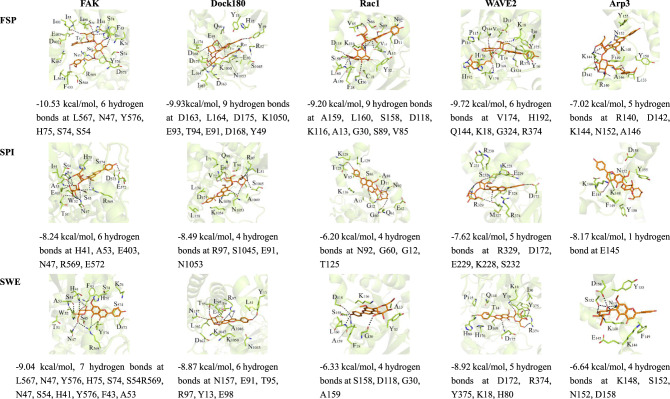
Interaction modes of 6'''-feruloylspinosin (FSP), spinosin (SPI), and swertisin (SWE) with the binding pocket of FAK, Dock180, Rac1, Arp3, WAVE2. The black-dashed lines indicate hydrogen bonds with the residues in the binding cavity of proteins.

The molecular docking energy scores and chemical structures of six in vivo SZS compounds are listed in Supplementary Table S4. Three in vivo SZS compounds (SPI, SWE, and FSP) showed better docking energy scores than COC, ZIZ, and SAP. FAK1, Dcok180, Rac1, and Arp3 were primarily targeted by FSP, SPI, and SWE. Thus, FSP, SPI, and SWE were selected for further molecular dynamics simulation. The results indicated that FSP, SPI, and SWE could be embedded into the canonical ligand-binding cavity of FAK, Dock180, Rac1, and Arp3. Of these compounds, FSP interacted with most of the target proteins with the lowest docking energies, followed by SWE. FSP, SPI, and SWE formed multiple hydrogen bonds with a few amino acid residues in FAK, Dock180, Rac1, and Arp3 (shown in Fig. 5). SWE showed stronger binding activity with FAK compared with the other proteins. SPI exhibited strong interaction with DOCK180 among the five signaling pathway proteins. FSP formed nine hydrogen bonds with the specific amino acid residues in DOCK180 and Rac1. SWE and SPI formed seven and six hydrogen bonds with specific residues in FAK, respectively. These residues may represent target sites for SPI, SWE, FSP. Compared with other proteins, because the structure of WAVE2 was narrow and long, it could not form a good binding cavity structure. As a result, three SZS in vivo compounds had a relatively lower binding affinity with WAVE2.

## Discussion

In the present study, we determined the protective effects of SZS on BBB injury induced by LPS. Changes in the protein profile of rat cortex with SZS and LPS treatment were established using proteomics, which provides a new perspective for understanding BBB integrity and protection. The dose of SZS decoction used for the rat studies was decided by our previous study. Many studies have indicated that SZS has antioxidant effects, whereas most of them focused on in vitro antioxidant activity^8^. Here, we describe the in vivo antioxidant effects of SZS against LPS exposure through decreasing levels of ROS, MDA, and H_2_O_2_, and increasing SOD activity and GSH levels. TEM results indicated that SZS protects the mitochondrial structure. Proteomic revealed that several DEPs in the mitochondrial inner membrane were significantly regulated in LPS/CON and SZS/LPS comparison groups, including acyl-coenzyme A synthase long-chain member (ACSL5) and fumarylacetoacetate hydrolase domain containing 1 (Fahd1). ACSL5 is an important regulator of whole-body energy metabolism. Overexpression of ACSL5 increased fatty acid oxidation and free radical formation. Elevated ACSL5 expression increased mitochondrial reactive oxygen species (ROS) production in human skeletal muscle^20^. It has been demonstrated that ACSL5 is involved in pathways associated with amyotrophic lateral sclerosis (ALS)^21^. Petit et al. reported that depletion of Fahd1 in human endothelial cells inhibited mitochondrial electron transport and induced premature senescence^22^. SZS downregulated the expression of ACSL5 and Fahd1, which may enhance the antioxidant activity of SZS.

It has been reported that oxidative stress damages BBB integrity by degrading junction proteins^13^. Cell–cell junctions include adherens and tight junctions (AJs and TJs). TJs and AJs are physical connections that form the barrier between blood and tissue. TJs and AJs between the endothelial cells of the BBB maintain barrier function through multiprotein complexes, such as ZO-1, claudin-5, occludin, and E-cadherin^23^. TJs are associated with many neurodegenerative disorders^24^. Western blot analysis indicated that SZS increased the expression of ZO-1 and occludin in LPS-exposed rat brain and hCMEC/D3 cells. An immunofluorescence assay revealed that SZS reversed the redistribution of ZO-1 and E-cadherin, and elevated the expression of ZO-1 and E-cadherin in hCMEC/D3 cells. These results indicate that SZS restores TJs and AJs, eventually alleviating the BBB disruption induced by LPS. As a transporter in brain microvascular endothelial cells, P-gp expression or function may regulate BBB permeability by limiting the entry of toxins or exogenous substances into the brain. Abnormal expression or dysfunction of P-gp is related to many neurological diseases, including Alzheimer’s disease and amyotrophic lateral sclerosis^25^. LPS treatment significantly decreased P-gp expression, and SZS increased P-gp expression in the rat cortex and hCMEC/D3 cells. Elevated P-gp expression may be useful for maintaining brain barrier function and brain hemostasis.

In the present study, we elucidated the overall effect of LPS treatment on the rat cortex and the protective effect of SZS administration using a proteomic strategy. The proteomics results showed that LPS treatment affected cell adhesion, intermediate filament, and neurofilament cytoskeleton organization in rat brains. It increased annexin A1 (Anxa1) expression and decreased kinesin-1 heavy chain expression. Anxa1 can inhibit the expression of cyclooxygenase-2 (COX-2) in macula densa, inhibit cell proliferation, and cause aberrant dysregulation of cytoskeletal proteins^26^. Kinesin-1 can activate the motor for microtubule binding and motility^27^. In addition, some of the DEPs in the LPS/CON comparison group were associated with GDP and GTP binding, such as ras homolog family member Q (RhoQ). RhoQ is a member of the Rho GTPases, which regulate the cytoskeletal structure, cellular protrusion, and migration^28^. This suggests that LPS treatment disrupts the cell adhesion and cytoskeleton in the rat cortex, potentially resulting in increased BBB permeability. This was demonstrated by increased EB permeability and wider endothelial cell intercellular spaces in rat brains treated with LPS. After administration of SZS to rats, the abnormal expression of Anxa1, kinesin-1 heavy chain, and RhoQ induced by LPS was restored. SZS also regulates the expression of some cell junction related proteins, such as cysteine-rich PDZ-binding protein (Cript), signal-induced proliferation-associated 1-like protein 1 (Sipa1l1), and C-terminal-binding protein 2 (Ctbp2). Cript directly binds to microtubules, linking the microtubule cytoskeleton and PSD-95 family scaffold proteins, and increases the number of dendritic spines^29^. Studies have suggested that the loss of dendritic spines may contribute to memory, learning, and behavioral deficits in patients with neurodegenerative diseases^30^. The proteomics results also showed that SZS regulates the dendritic spine and post-synapses, which suggests that SZS be used to treat neurodegenerative diseases accompanied by decrease BBB permeability. Most studies have focused on inflammation through the NF-κB signaling pathway or oxidative damage by the Nrf2 pathway in BBB damage models induced by LPS^13^. The present study is the first to report global protein changes in the rat cortex following LPS exposure or SZS intervention, and provides comprehensive protein expression data and putative underlying mechanisms.

The present study demonstrates that SZS restores BBB damage induced by LPS by regulating cell adhesion, cell junctions, and the actin cytoskeleton, which is different from studies focused on the RhoA/ROCK, NF-κB, or Nrf2 signaling pathways. The Arp2/3 complex controls actin polymerization by assembling actin monomers (G-actin) into branched actin filament (F-actin) networks and plays an important role in cadherin/catenin complex-mediated endothelial cell–cell adhesion and the formation and maintenance of barrier integrity^31^. The Arp2/3 complex is activated by the WAVE regulatory complex downstream of Rac1 GTPase^17^. The FAK-DOCK180 signaling cascade plays an important role in the activation of Rac1 GTPase^19^. The FAK-DOCK180-Rac1-WAVE2-Arp3 signaling pathway may play an important role in the integrity of the BBB by regulating the polymerization and depolymerization of F-actin. We demonstrated that SZS protected LPS-induced BBB damage by FAK-DOCK180-Rac1-WAVE2-Arp3 signaling to increase the ratio of F-actin to G-actin in the rat cortex and hCMEC/D3 cells, thus protecting BBB integrity.

The chemical components of SZS are the therapeutic material basis for SZS to exhibit pharmacodynamic effect. It is necessary to study the chemical components of SZS in vivo. It is necessary to study the chemical components of SZS in vivo. Therefore, the components in SZS decoction and the plasma of rats administrated with SZS decoction were identified by HPLC-HR-MS. We identified 33 chemical constituents in SZS decoction by HPLC-HR-MS, including SWE, SPI, FSP, ferulic acid (FA), and jujuboside B (JUB). SWE, SPI, FSP, isocorydine (ICD), NUC, FA, and JUB were also quantified with commercially available standards by HPLC-triple quadrupole tandem MS. The contents of SWE, SPI, FSP, ICD, NUC, FA, and JUB were 0.0135%, 0.685%, 0.093%, 0.128%, 0.096%, 0.126%, 0.041%, respectively. It is believed that the absorbed components of medicinal plants may be potentially active constituents; thus, the SZS components in the plasma of rats administered SZS decoction were further analyzed by HPLC-HR-MS. Six compounds, including SPI, FSP, SWE, COC, ZIZ, and SAP, were identified and confirmed with standards. Jujubosides in SZS are some of the active components which exert hypnotic, antioxidant, and neuroprotective effects^32^. However, SZS jujubosides exhibit relatively low bioavailability because they are metabolized by stomach acid or gut flora^33^. JUB was not identified in rat plasma in the present study. These six in vivo components may contribute to BBB protection. In order to screen interaction, binding mode, potential binding sites between in vivo components with potential targets, molecular docking between in vivo components and pathway proteins (FAK, DOCK180, Rac1, WAVE2, and Arp3) was performed. FSP, SPI, and SWE exhibited lower docking energies compared with COC, ZIZ, and SAP. Molecular dynamic simulation indicated that FSP strongly binds to FAK, followed by DOCK180 and Rac1. SWE and SPI bind to FAK and DOCK180 with the lowest docking energies. Figure 5 shows that SPI, SWE, and FSP bind to proteins at multiple amino acid residues; however, whether these residues are potential target sites of SPI, FSP, and SWE requires further study. Available reports have demonstrated that SPI, FSP, and SWE may attenuate Alzheimer’s disease-associated synaptic dysfunction, alleviate beta-amyloid induced toxicity, inhibit Aβ 1-42 production and aggregation, and improve cognitive impairment in mouse and cell models^34,35^. Our molecular docking results indicate that SPI, FSP, and SWE potentially interact with FAK, DOCK180, Rac1, WAVE2, and Arp3, which supports the concept that SPI, FSP, and SWE are the active constituents in SZS that protect the BBB. This result seems to correspond to available reports that SPI, FSP, SWE are the active compounds to prevent AD or other central nervous diseases^10^. However, protective effect on BBB of these compounds needs to be further validated with animal or cell models.

In conclusion, this is the first report to demonstrate that SZS prevents LPS-induced BBB dysfunction by up-regulating the FAK-DOCK180-Rac1-WAVE2-Arp3 pathway based on proteomics and biochemical assays. Our findings suggest that SZS is a potentially neuroprotective food that acts upon the BBB. The present study provides novel insights into the beneficial effects of SZS against BBB-related neurological diseases. Furthermore, it enables a better understanding of the underlying mechanism of the neuroprotective effects of SZS and supports the use of SZS as a functional food and medicinal plant.

## Methods

### Chemicals and reagents

SZS was purchased from the Beijing Tongrentang Pharmaceutical Co. Ltd. (Beijing, China) and identified as dry mature seeds of the rhamniaceae plant *Ziziphus jujuba* Mill. Var. *spinosa* (Bunge) Hu ex H.F. Chou. by Dr. Yujuan Li (School of Life Sciences, Beijing Institute of Technology). The specimen (No. 20201015) was preserved in the School of Life Science, Beijing Institute of Technology. SZS was boiled two times with distilled water (1 h each time) to prepare the SZS decoction (0.2 g/mL). Vitamin C tablets (Vc, Lot No. 20200635, Huazhong Pharmaceutical Co. Ltd., Hubei, China) were dissolved to prepare a solution (20 g/mL) with deionized water. Lipopolysaccharide (LPS, Lot No. 325D037) was purchased from Solarbio Science & Technology Co., Ltd. (Beijing, China).

Rabbit polyclonal antibodies against occludin, β-catenin, FAK, Arp3, P-gp, goat polyclonal antibody against ZO-1 and mouse polyclonal antibody against Rac1, E-cadherin were purchased from Abcam (Cambridge, MA, USA). Rabbit polyclonal antibodies against DOCK180 and WAVE2 were obtained from Cell Signaling Technology (Danvers, MA, USA) and ImmunoWay Biotechnology Co. (Plano, TX, USA), respectively. G-actin: F-actin in vivo assay kit was purchased form Cytoskeleton Inc. (Denver, CO, USA). Horseradish peroxidase (HRP)-conjugated secondary antibodies, including anti-rabbit, anti-mouse, and anti-goat IgG H&L, were obtained from the Dakemei Technology Company (Shanghai, China). ROS, PCB, SOD, GSH, H2O2, MDA assay kits (category number: E004-1-1, A087-1-2, A001-3-2, A006-2-1, A064-1-1 and A003-4-1) were obtaind from Nanjing Jiancheng Bioengineering Institute (Jiangsu, China). Rat and Human TNF-α and IL-6 ELISA kits (category number: YM-S1716, YM-S1726, YM-S0122 and YM-S0049) were purchased from Shanghai Yuanmu biotech Co., Ltd. (Shanghai, China). The TUNEL apoptosis kit was provided by Servicebio Co. (Hubei, China).

### Animal treatment and sample collection

Forty Sprague-Dawley (SD) rats (half male and female, weighing 200 ± 20 g) were purchased from the Beijing HFK Bioscience Co. Ltd (Beijing, China). All experimental procedures involving animals complied with the Guide for the Care and Use of Laboratory Animals published by the National Institutes of Health (NIH publication No.85-23, revised in 1996) and were approved by the Beijing Institute of Technology Animal Care and Use Committee (SYXK-BIT-20200109002, August 2020). Rats were maintained in the same environment with a 12 h light/dark cycle and a relative humidity of 40–70% at 24 ± 2 °C. Rats had free access to water and food. All rats were acclimatized for one week and then randomly divided into CON, LPS, SZS, and positive control (POS) groups.

SZS and POS groups rats were, respectively, administered SZS (2 g/kg) and V_C_ (0.2 g/kg) orally once daily for 30 days. Equal volumes of distilled water were ingested by the CON and LPS groups. The rats, except the CON group, were injected intraperitoneally (i.p.) with LPS (10 mg/kg), and after the last dose, the rats were fasted overnight with free access to water. 12 h after LPS treatment, the rats were anesthetized with chloral hydrate (350 mg/kg, i.p.). Blood samples were collected from the heart into heparinized tubes and centrifuged to obtain plasma. After perfusion through the rat heart with pre-cooled saline, the rat brain was immediately collected on ice. One hemibrain from each group was immersed in 4% paraformaldehyde for H&E, Nissl, and TUNEL staining. Approximately 0.5 cm^3^ cerebral cortex from the other hemibrain was fixed in 2.5% glutaraldehyde for TEM observation. The remaining samples were stored at −80 °C for further analysis.

### Cell culture

The immortalized human brain endothelial capillary cell line, hCMEC/D3, was cultured in RPMI 1640 medium (Gibco, Invitrogen, Paisley, UK) supplemented with 10% fetal bovine serum (Solarbio, Beijing, China), 1% penicillin and streptomycin (Solarbio, Beijing, China) at 37 °C in a humidified incubator with 5% CO_2_. The cells were divided into six groups, including CON, LPS, low-dose SZS (L-SZS), middle-dose SZS (M-SZS), high-dose SZS (H-SZS) and POS groups. Cells in all groups were cultured under the same culture conditions. Cells in L-SZS, M-SZS, H-SZS and POS groups were preincubated with SZS decoction (50, 100, 200 μg/mL) and Vc (50 μg/mL) for 24 h, respectively. Then, all groups, except the CON group, were treated with 10 μg/mL of LPS for an additional 24 h. After washing with PBS, the cell pellets were collected by centrifugation.

### Histopathological evaluation and flow cytometry analysis

The rat hemibrain fixed in 4% paraformaldehyde was paraffin-embedded, cut into sections, and stained with H&E using routine procedures^36^. Nissl and TUNEL staining were performed as previously described^37^. Histological images were captured with a Nanozoomer S210 microscopic-resolution scanner equipped with Digital Pathology View 2.0 software (Hamamatsu Photonics, Shizuoka, Japan). Flow cytometry analysis of apoptotic cells was done using an FITC Annexin-V Apoptosis Detection Kit (Beyotime) according to the manufacturer’s instructions.

### TEM observation

Ultrathin sections of the cerebral cortex fixed in 2.5% glutaraldehyde were prepared following published methods^38^. Briefly, samples were dehydrated in a graded series of ethanol, embedded in eponate 812, and cut into ultrathin sections. The sections were stained with uranyl acetate and lead citrate and examined with a JEM-1400 electron microscope (JEOL Ltd., Tokyo, Japan).

### Permeability determination

The EB dye was used to evaluate the permeability of the BBB. Rats were intravenously injected with 2% EB (2 mg/kg) through the tail vein 90 min before they were sacrificed. After the rat brains were collected and imaged, brain samples were weighed and homogenized. The EB fluorescence intensity was determined using a Cytation/3 imaging reader (BioTek, Vermont, VT, USA) at the excitation and emission wavelengths of 620 and 680 nm, respectively.

The paracellular permeability of the hCMECs/D3 monolayer was evaluated by the diffusion of sodium fluorescein^13^. Briefly, 1.5 mL of HBSS was added to the lower compartment of a Transwell chamber, and 0.5 mL of fluorescein sodium (500 μg/mL) was added to the upper chamber and incubated at 37 °C for 1 h in the dark. Then, 100 μL of sample from the lower chamber was collected and the absorbance was measured at 492 nm.

### Trans-endothelial electrical resistance (TEER) measurement

The TEER value was used to evaluate the integrity of the hCMECs/D3 monolayer with an epithelial-volt-ohm resistance meter (RE1600, Kingtech, Beijing, China). Briefly, cell inoculation was done as previously described^39^. When TEER values reached a peak, the cells were incubated with SZS and Vc, followed by LPS. TEER values (Ω cm^2^) were determined as previously described (Li et al., 2018).

### Biochemical assays

Homogenates of rat cortex and hCMECs/D3 cells were prepared and centrifuged. Supernatants were collected for biochemical assays. The contents of TNF-α and IL-6 in cells and rat cerebral cortex and plasma were determined with an ELISA kit according to the manufacturer’s instructions. The levels of MDA, GSH, H_2_O_2_, ROS, PCB, and SOD activity in rat cortex and plasma were determined using commercial kits following the manufacturer’s protocol.

### Western blot analysis

Samples of rat brain and cell pellets were homogenized using radio immunoprecipitation assay (RIPA) lysis buffer. The homogenates were centrifuged and the supernatant was collected. Equal amounts of each protein sample were separated by sodium dodecyl sulfate-polyacrylamide gel electrophoresis (SDS-PAGE) and transferred to poly vinyli dene fluoride (PVDF) membranes. The membranes were incubated with primary antibody overnight at 4 °C followed by a secondary antibody for 2 h at room temperature. The dilutions of the primary antibodies for ZO-1, occludin, β-catenin, E-cadherin, P-gp, Rac1, WAVE2, and Arp3 were 1:5000. For FAK and DOCK180, the dilutions were 1:1000 and 1:2000, respectively.

### Immunofluorescence assay

The hCMEC/D3 cells treated with SZS or LPS and grown on a confocal plate were fixed, permeabilized, and blocked according to routine procedures. For F-actin staining, the cells were incubated with phalloidin (1:200). For staining junction proteins, the cells were incubated with anti-occludin (1:200), anti-E-cadherin (1:200), or anti-ZO-1 (1:400) antibodies. The cells were then treated with TRITC-conjugated secondary antibodies (1:100) and 4′, 6-diamidino-2-phenylindole (1:100, DAPI). Images were captured using a Nikon N-SIM confocal microscope (Nikon, Tokyo, Japan).

### Proteomics

Six biological replicates (rat cortex) in the CON, LPS, and SZS groups were separately homogenized with lysis buffer. Protein (200 μg) from each sample was digested with trypsin. Briefly, the steps included protein denaturation, disulfide bridge reduction, alkylation, trypsin digestion, and termination^40^. The samples were desalted with a MonoSpin C_18_ desalting column (GL Science, Tokyo, Japan). Finally, the dried peptides were stored at −80 °C until use.

Each peptide sample (200 ng) was separated using NanoElute LC coupled with trapped ion mobility-quadrupole time-of-flight (TIMS-TOF) MS and a modified nano-electrospray ion source (Bruker Daltonics, Billerica, MA, USA). A C_18_ reverse-phase column (3 µm, 250 mm × 75 µm, Ion Opticks, Parkville, Australia) was selected as the stationary phase. Mobile phase A contained 0.1% formic acid in water, and mobile phase B was acetonitrile. The flow rate of the mobile phase was 300 nL/min. The separation gradient was set as follows: 0–45 min, 2–22% B; 45–50 min, 22–37% B; 50–55 min, 37–80% B; 55–60 min, 80% B. The scanning range of mass was from 100 to 1700 m/z, and the ion mobility was scanned from 0.7 to 1.3 Vs/cm^2^. The acquisition time of a single cycle was 1.16 s. The accumulation and ramp time were set to 100 ms each. The voltage of the ion source was 1500 V. The temperature of the ion source was set to 180°C, and the flow rate of the auxiliary gas was maintained at 3 L/min.

Peaks online software (Bioinformatics Solutions Inc, Waterloo, Canada) and the Swiss-port database were used to identify proteins. The parameters were as follows: the species was Rattus norvegicus, the precursor mass error tolerance was 15 ppm, the fragment mass error tolerance was 0.05 Da, and the digestion mode for the trypsin enzyme was set up as semi-specific. The fixed modification was carbamidomethylation, and alterable modifications included acetylation, oxidation, and deamidation. Protein abundance ratios of LPS to CON or SZS to LPS were defined as fold-change. DEPs were designated if the fold change was >1.4 or <0.71 with a P value less than 0.05. All DEPs were functionally annotated and clustered using the Database for Annotation, Visualization, and Integrated Discovery (DAVID) available at http://david.ncifcrf.gov (version 6.8).

### UPLC-HR-MS for the identification of SZS constituents in SZS decoction and rat plasma

The ultimate 3000 hyperbaric LC system coupled with high-resolution Orbitrap Fusion Lumos Tribrid via an electrospray ionization (ESI) interface (UPLC-HR-MS, Thermo Fisher Scientific, Bremen, Germany) was used to comprehensively analyze the prototypes and metabolites of SZS in rat plasma and the components in the SZS decoction. The chromatography system was equipped with an auto-sampler, a diode-array detector, a column compartment, and two pumps. The chromatographic conditions were optimized, and a BEH C_18_ column (1.7 μm, 2.1 mm × 100 mm, Waters, MA, USA) maintained at 35 °C was selected to separate the SZS constituents. The mobile phase consisted of 0.1% formic acid in water A) mixed in a gradient mode with acetonitrile plus 0.1% formic acid, B) at a flow rate of 200 μL/min. The elution gradient was optimized as follows: 0–2 min, 3% B; 2–5 min, 3–45% B; 5–20 min, 45–75% B; 20–24 min, 75% to 100% B; and 24–32 min, 100% B. The injection volume was 3.0 μL and the sampler was maintained at 4 °C.

The identification of SZS components in rat plasma (in vivo) and SZS decoction was made in positive full scan modes within the range of mass/charge ratio 150–1500 at a resolution of 120,000 for the acquisition of accurate molecular ions. The other parameters were set as follows: spray voltage, +3.0 kV; sheath gas flow rate, 35 arb; aux gas flow rate, 10 arb; sweep gas, 2 arb; capillary temperature, 325 °C; vaporizer temperature, 275 °C; RF lens, 50%; AGC target, 400000. The fragment ions in MS/MS data obtained by higher energy collision dissociation at proper collision energy were further used to confirm the structures of the in vivo SZS components. In addition, JUB, SPI, SWE, FSP, NUC, COC, and ICD standards were used for the identification of the components. Xcalibur 3.0 software (Thermo Fisher Scientific, MA, USA) was used for UPLC-HR-MS control and data handling.

### Molecular docking analysis

In vivo SZS components were selected for further interaction with proteins related to BBB damage (junction and potential signaling pathway proteins) using the Libdock protocol in AutoDock 4.2 (www.bioms.org). Docking scores were obtained as described elsewhere^15^. Further, a molecular dynamics simulation analysis between the potential signaling pathway proteins and the in vivo SZS compounds with the lowest docking energy was performed.

### Statistical analysis

Data were expressed as means ± SD. The mean differences between multiple groups were compared using a one-way analysis of variance, and *P* values less than 0.05 were considered statistically significant.

### Reporting summary

Further information on research design is available in the Nature Research Reporting Summary linked to this article.

## Supplementary information

Supplementary File

Full scan images

Nr-Reporting-Summary

## Data Availability

The data supporting the findings reported here are available on reasonable request from the corresponding author.
