# Supplementary material for: Semen Ziziphi Spinosae attenuates blood–brain barrier dysfunction induced by lipopolysaccharide by targeting the FAK-DOCK180-Rac1-WAVE2-Arp3 signaling pathway
Source: NPJ Sci Food. 2022 Jun 2;6:27. doi: 10.1038/s41538-022-00142-6 (PMC9163036; doi:10.1038/s41538-022-00142-6)
Supplement: Supplementary file 1 — Supplementary File [file 41538_2022_142_MOESM1_ESM.pdf]

**Rat brain**

ZO-1

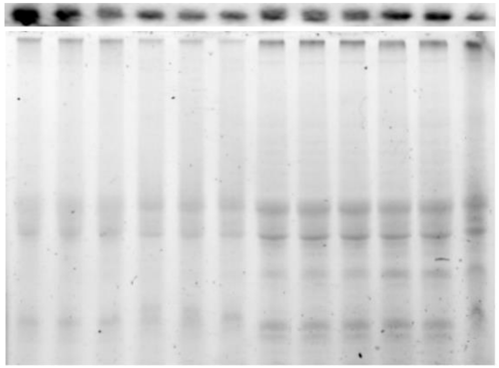

Occludin

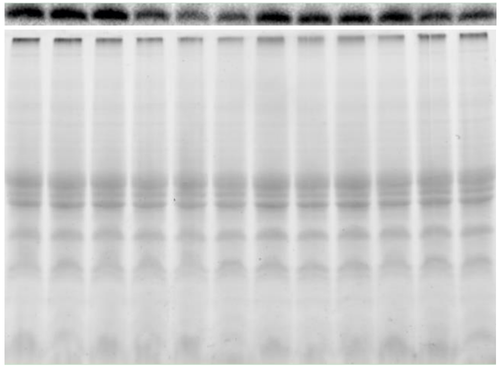

E-cadherin

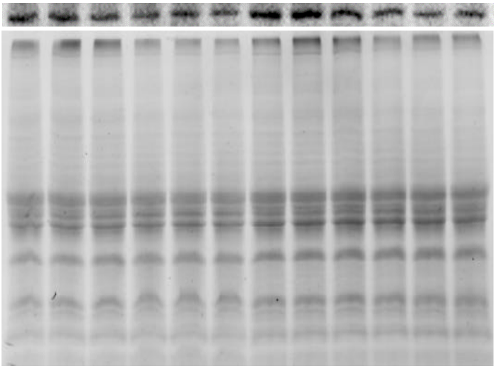

β-catenin

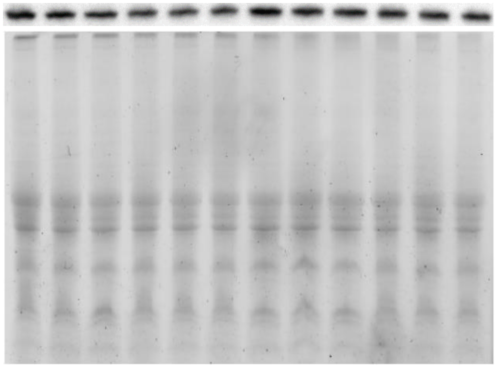

P-gp

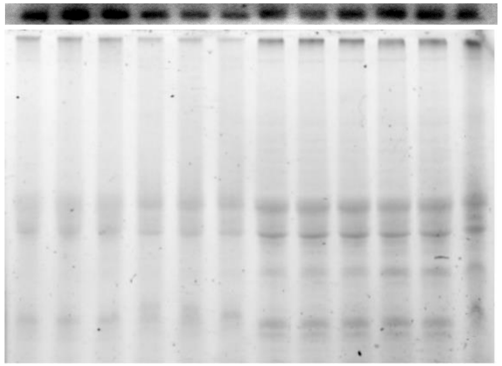

**hCMEC/D3 cell**

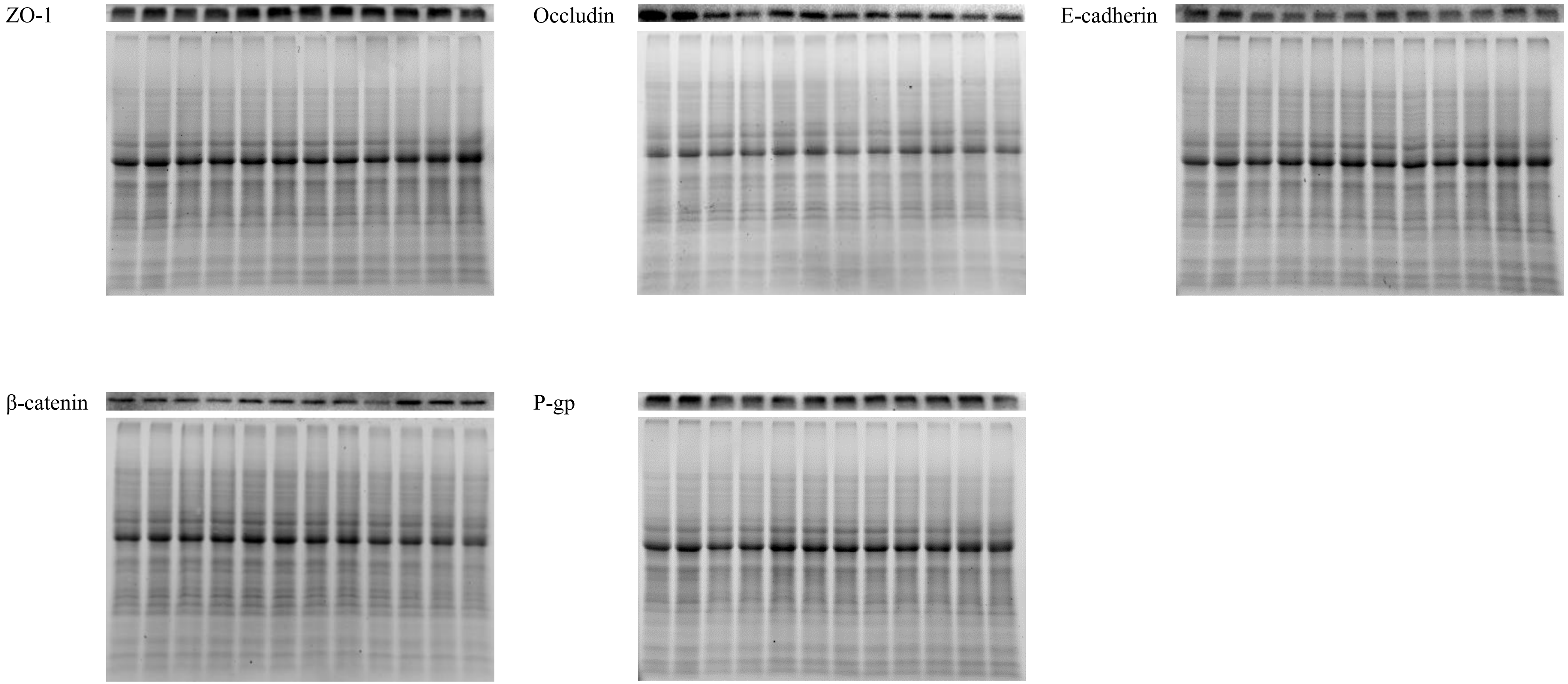


**Supplementary Figure 1** Western-blot bands of TJ, AJ proteins and P-gp in rat brain and hCMEC/D3 cell, and their corresponding total protein gels.

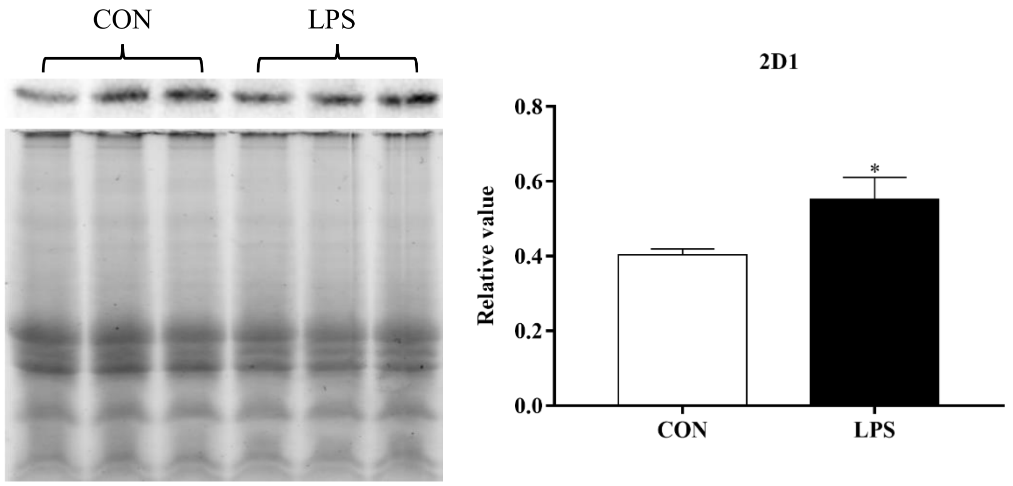


**Supplementary Figure 2** The expression of cytochrome P450 2D1 (CYP2D1) by Western-blot method to demonstrate a pattern consistent with MS analysis.

**Rat brain**

FAK

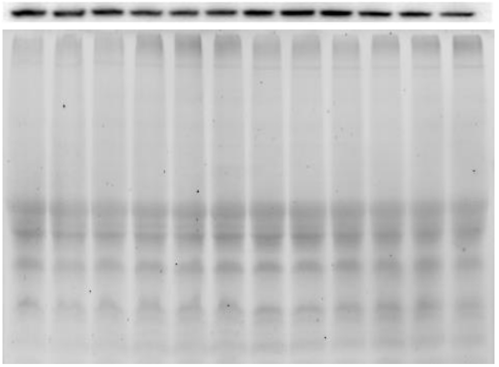

DOCK180

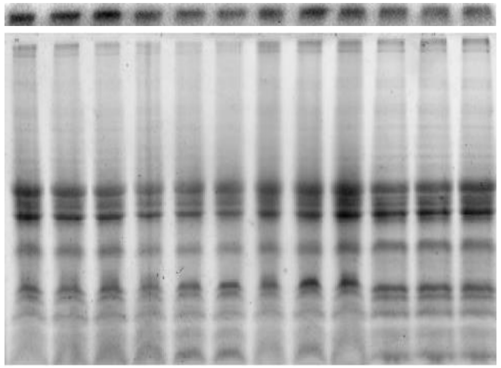

Rac1

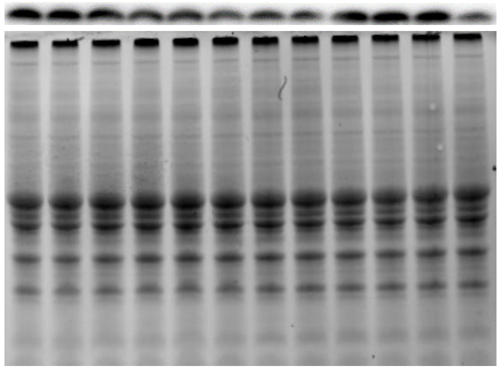

WAVE2

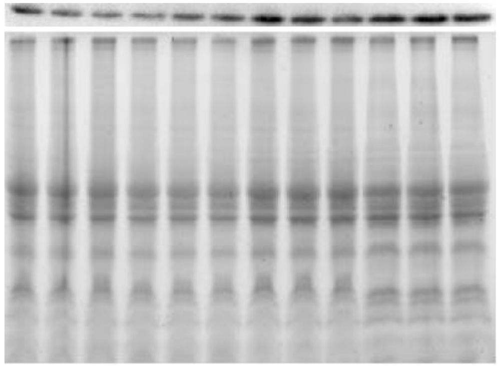

Arp3

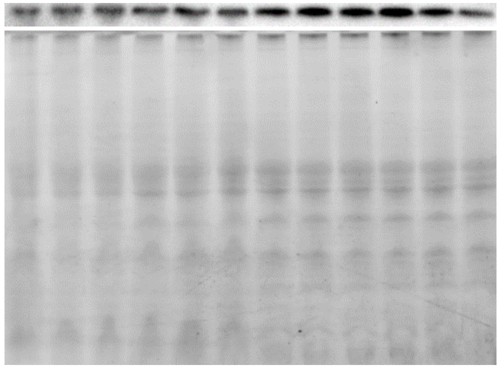

**hCMEC/D3 cell**

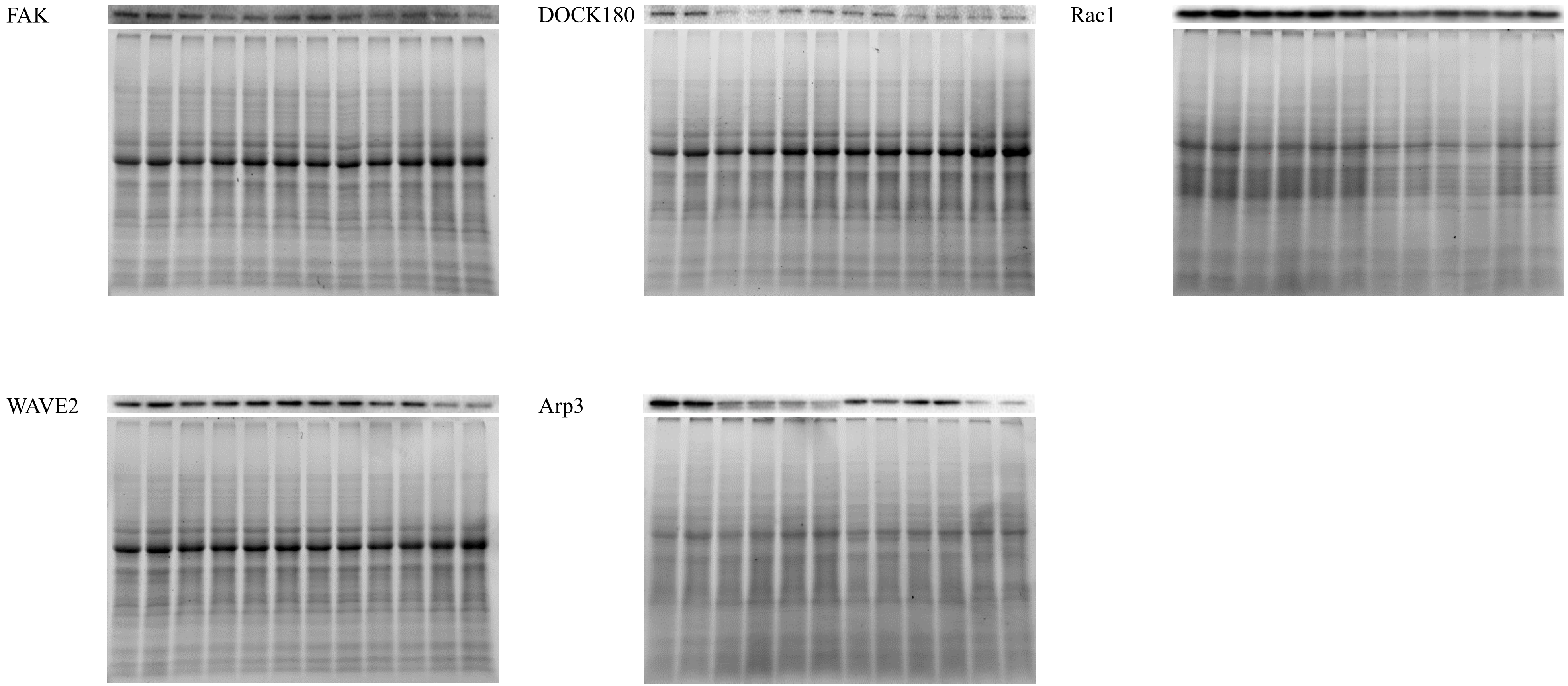


**Supplementary Figure 3** Western-blot bands of FAK, DOCK180, Racl, WAVE2, Arp3 and its corresponding total protein gels in rat brain and hCMEC/D3 cell.

**Supplementary Table 1** The list of all differently expressed proteins.

| LPS/CON comparison group | | | | |
|---|---|---|---|---|
| Gene IDs | Protein names | Fold change | | *p*-value |
| Q641Y7 | 8-oxo-dGDP phosphatase NUDT18 | 0 | ↓ | 0.016207 |
| Q6P9V9 | Tubulin alpha-1B chain | 0.133855352 | ↓ | 0.00202 |
| P68035 | Actin, alpha cardiac muscle 1 | 0.149921289 | ↓ | 0.045649 |
| Q63258 | Integrin alpha-7 | 0.218226207 | ↓ | 0.015461 |
| P62986 | Ubiquitin-60S ribosomal protein L40 | 0.270674389 | ↓ | 0.042454 |
| Q3B7K9 | RUN domain-containing protein 3B | 0.285826895 | ↓ | 0.001883 |
| Q9WVC1 | Slit homolog 2 protein (Fragment) | 0.312321196 | ↓ | 0.045786 |
| P21263 | Nestin | 0.322180432 | ↓ | 0.000148 |
| Q04400 | Adenylate cyclase type 5 | 0.362605465 | ↓ | 0.007001 |
| F1LM93 | Tyrosine-protein kinase Yes | 0.399736211 | ↓ | 0.037261 |
| Q03626 | Murinoglobulin-1 | 0.407237806 | ↓ | 0.000631 |
| Q2PQA9 | Kinesin-1 heavy chain | 0.407561905 | ↓ | 0.008357 |
| Q8CIN9 | E3 ubiquitin-protein ligase rififylin | 0.408553557 | ↓ | 0.028558 |
| Q5RJN4 | Shiftless antiviral inhibitor of ribosomal frameshifting protein homolog | 0.416151846 | ↓ | 0.013172 |
| Q52KK4 | H/ACA ribonucleoprotein complex non-core subunit NAF1 | 0.417927438 | ↓ | 0.016672 |
| Q9QZ76 | Myoglobin | 0.420183533 | ↓ | 0.032707 |
| Q63648 | Merlin (Fragment) | 0.427279107 | ↓ | 0.009307 |
| P59824 | Interleukin-1 receptor accessory protein-like 1 | 0.447973104 | ↓ | 0.012547 |
| Q63511 | G protein-activated inward rectifier potassium channel 3 | 0.449430355 | ↓ | 0.002417 |
| P08733 | Myosin regulatory light chain 2, ventricular/cardiac muscle isoform | 0.461843425 | ↓ | 0.002173 |
| Q4QQT5 | Probable isocitrate dehydrogenase [NAD] gamma 2, mitochondrial | 0.47635222 | ↓ | 0.022204 |
| Q5RKH6 | Protein OS-9 | 0.487947133 | ↓ | 0.001586 |
| Q925C0 | Synaptotagmin-9 | 0.492370436 | ↓ | 0.004913 |

Continue Table

| Gene IDs | Protein names | Fold change | | p-value |
|---|---|---|---|---|
| Q02356 | AMP deaminase 2 | 0.492724837 | ↓ | 0.036227 |
| Q01177 | Plasminogen | 0.497541039 | ↓ | 0.00098 |
| Q9JJS5 | SH3 domain-binding protein 4 | 0.500175243 | ↓ | 0.010116 |
| Q5FVG2 | Band 4.1-like protein 5 | 0.517260865 | ↓ | 0.042755 |
| Q6DTM3 | Jouberin | 0.521995082 | ↓ | 0.024529 |
| P13832 | Myosin regulatory light chain RLC-A | 0.52715689 | ↓ | 0.000693 |
| Q5XHX6 | Thioredoxin domain-containing protein 2 | 0.532935168 | ↓ | 0.023531 |
| P50237 | Sulfotransferase 1C1 | 0.53478231 | ↓ | 0.005402 |
| P55016 | Solute carrier family 12 member 1 | 0.541915081 | ↓ | 0.00606 |
| P50617 | Dendrin | 0.549152891 | ↓ | 0.010282 |
| P07092 | Glia-derived nexin | 0.553594597 | ↓ | 0.000369 |
| Q66H50 | Fatty acyl-CoA reductase 1 | 0.559418586 | ↓ | 0.002196 |
| Q5XI03 | Centrosomal protein of 95 kDa | 0.56179938 | ↓ | 0.013947 |
| Q6TLK4 | Rho GTPase-activating protein 27 | 0.572587426 | ↓ | 0.016003 |
| Q6REY9 | Rho GTPase-activating protein 20 | 0.576921653 | ↓ | 0.003387 |
| P70507 | G protein-coupled receptor kinase 4 | 0.58267248 | ↓ | 0.001605 |
| Q62761 | Casein kinase I isoform gamma-1 | 0.586078272 | ↓ | 0.017523 |
| O88368 | Microphthalmia-associated transcription factor | 0.590204547 | ↓ | 0.025499 |
| B4F7E8 | Protein Niban 2 | 0.600868975 | ↓ | 0.020423 |
| Q62925 | Mitogen-activated protein kinase kinase kinase 1 | 0.601417848 | ↓ | 0.046047 |
| P60571 | Pannexin-2 | 0.602333201 | ↓ | 0.002625 |
| Q9Z221 | Polyamine-modulated factor 1-binding protein 1 | 0.602822731 | ↓ | 0.001122 |
| P50123 | Glutamyl aminopeptidase | 0.610203999 | ↓ | 0.003454 |
| Q5M7A3 | Solute carrier family 35 member G2 | 0.612374735 | ↓ | 0.012519 |

Continue Table

| Gene IDs | Protein names | Fold change | | p-value |
|---|---|---|---|---|
| P17178 | Sterol 26-hydroxylase, mitochondrial | 0.612471856 | ↓ | 0.002105 |
| Q5M9F0 | UPF0705 protein C11orf49 homolog | 0.614005876 | ↓ | 0.011625 |
| P24062 | Insulin-like growth factor 1 receptor | 0.617153191 | ↓ | 0.015722 |
| P82458 | E3 ubiquitin-protein ligase Midline-1 | 0.617913322 | ↓ | 0.009214 |
| D3ZEN0 | MICAL-like protein 2 | 0.618461918 | ↓ | 0.008403 |
| Q9R237 | C-Jun-amino-terminal kinase-interacting protein 1 | 0.620101967 | ↓ | 0.009522 |
| P62982 | Ubiquitin-40S ribosomal protein S27a | 0.628185772 | ↓ | 0.01756 |
| D4A631 | Brefeldin A-inhibited guanine nucleotide-exchange protein 1 | 0.639441872 | ↓ | 0.036729 |
| Q63149 | Cadherin-4 (Fragment) | 0.645630577 | ↓ | 0.009147 |
| P14659 | Heat shock-related 70 kDa protein 2 | 0.64845663 | ↓ | 0.022218 |
| P07882 | Bile salt-activated lipase | 0.649095926 | ↓ | 0.001354 |
| Q5SGD7 | Connector enhancer of kinase suppressor of ras 3 | 0.650772888 | ↓ | 0.003252 |
| A1A5P9 | Melanoma-associated antigen E1 | 0.650844869 | ↓ | 0.012695 |
| P80204 | TGF-beta receptor type-1 | 0.660414639 | ↓ | 0.022782 |
| P70539 | Activin receptor type-1C | 0.660414639 | ↓ | 0.010814 |
| Q5U204 | Ragulator complex protein LAMTOR3 | 0.666453372 | ↓ | 0.033366 |
| O08700 | Vacuolar protein sorting-associated protein 45 | 0.672373827 | ↓ | 0.032902 |
| P55926 | Acid-sensing ion channel 1 | 0.679244316 | ↓ | 0.000123 |
| Q6AZ61 | Lysosomal cobalamin transport escort protein LMBD1 | 0.679853671 | ↓ | 0.002388 |
| Q9QXU8 | Cytoplasmic dynein 1 light intermediate chain 1 | 0.684044863 | ↓ | 0.008735 |
| Q66H39 | ATP-binding cassette sub-family F member 3 | 0.684102025 | ↓ | 0.028809 |
| Q9R095 | Sperm flagellar protein 2 | 0.685356725 | ↓ | 0.023437 |
| P62828 | GTP-binding nuclear protein Ran | 0.68650115 | ↓ | 0.00306 |
| P0DSP1 | Sortilin-related receptor | 0.68785998 | ↓ | 0.02335 |

Continue Table

| Gene IDs | Protein names | Fold change | | *p*-value |
|---|---|---|---|---|
| Q63366 | Neurexophilin-1 | 0.689310883 | ↓ | 0.021408 |
| Q64595 | cGMP-dependent protein kinase 2 | 0.691239176 | ↓ | 0.04997 |
| Q9Z1J8 | SEC14-like protein 3 | 0.691433903 | ↓ | 0.02099 |
| Q63164 | Dynein heavy chain 1, axonemal | 0.692629423 | ↓ | 0.029952 |
| P70569 | Unconventional myosin-Vb | 0.693603138 | ↓ | 0.002988 |
| Q7TQ20 | DnaJ homolog subfamily C member 2 | 0.695162318 | ↓ | 0.00051 |
| P52632 | Signal transducer and activator of transcription 5B | 0.696826008 | ↓ | 0.00419 |
| Q4QQT4 | Serine/threonine-protein phosphatase 2A 65 kDa regulatory subunit A beta isoform | 0.697035096 | ↓ | 0.006971 |
| P61354 | 60S ribosomal protein L27 | 0.697678917 | ↓ | 0.047171 |
| Q5U316 | Ras-related protein Rab-35 | 0.698269469 | ↓ | 0.002628 |
| Q64568 | Plasma membrane calcium-transporting ATPase 3 | 0.700290313 | ↓ | 0.001522 |
| Q9Z2Q4 | Methionine synthase | 0.702300989 | ↓ | 0.029 |
| Q811U3 | ELKS/Rab6-interacting/CAST family member 1 | 0.70335508 | ↓ | 0.008108 |
| Q5PPN4 | Carbonic anhydrase-related protein | 0.704084577 | ↓ | 0.000184 |
| O54960 | Carboxyl-terminal PDZ ligand of neuronal nitric oxide synthase protein | 0.704099618 | ↓ | 0.021916 |
| Q8K1Q4 | Leucine zipper putative tumor suppressor 3 | 0.711060609 | ↓ | 0.015089 |
| P86182 | Coiled-coil domain-containing protein 22 | 1.400854567 | ↑ | 0.001418 |
| Q6P6R7 | Small G protein signaling modulator 3 | 1.401021059 | ↑ | 0.026419 |
| Q05962 | ADP/ATP translocase 1 | 1.401119271 | ↑ | 0.000706 |
| Q5U2Y3 | MAGUK p55 subfamily member 7 | 1.402317933 | ↑ | 0.0306 |
| P23565 | Alpha-internexin | 1.402402972 | ↑ | 0.007165 |
| Q6P767 | Pituitary tumor-transforming gene 1 protein-interacting protein | 1.402527186 | ↑ | 0.008607 |
| Q5U2N3 | Membrane-associated phosphatidylinositol transfer protein 1 | 1.405724231 | ↑ | 0.020235 |
| P19234 | NADH dehydrogenase [ubiquinone] flavoprotein 2, mitochondrial | 1.406334135 | ↑ | 0.00015 |

Continue Table

| Gene IDs | Protein names | Fold change | | p-value |
|---|---|---|---|---|
| P63031 | Mitochondrial pyruvate carrier 1 | 1.406623918 | ↑ | 0.019505 |
| O88871 | Gamma-aminobutyric acid type B receptor subunit 2 | 1.406637182 | ↑ | 0.04899 |
| Q5I0K8 | 28S ribosomal protein S7, mitochondrial | 1.407492231 | ↑ | 0.03266 |
| Q5U1Z2 | Trafficking protein particle complex subunit 3 | 1.407773996 | ↑ | 0.005606 |
| P61212 | ADP-ribosylation factor-like protein 1 | 1.40904417 | ↑ | 0.005316 |
| Q8VHV7 | Heterogeneous nuclear ribonucleoprotein H | 1.40919758 | ↑ | 0.029721 |
| Q6GQP4 | Ras-related protein Rab-31 | 1.409565829 | ↑ | 0.005013 |
| Q63686 | Cyclin-dependent kinase 16 | 1.410457342 | ↑ | 0.022179 |
| P24368 | Peptidyl-prolyl cis-trans isomerase B | 1.413340024 | ↑ | 0.028275 |
| B4F7C5 | Leucine-rich repeat transmembrane neuronal protein 4 | 1.413459141 | ↑ | 0.005099 |
| Q6E0V2 | Katanin p60 ATPase-containing subunit A1 | 1.414301843 | ↑ | 0.04687 |
| D4ACN8 | Plasminogen receptor (KT) | 1.414685686 | ↑ | 0.047526 |
| O35052 | Phosphatidate cytidylyltransferase 1 | 1.417364352 | ↑ | 0.016274 |
| Q9EPB1 | Dipeptidyl peptidase 2 | 1.417428385 | ↑ | 0.013857 |
| P83565 | 39S ribosomal protein L40, mitochondrial | 1.417507915 | ↑ | 0.001505 |
| Q4KLK9 | RNA polymerase II subunit A C-terminal domain phosphatase SSU72 | 1.417794241 | ↑ | 5.65E-05 |
| Q6AXN3 | Transmembrane emp24 domain-containing protein 5 | 1.41783268 | ↑ | 0.007728 |
| Q9Z1C8 | Rap guanine nucleotide exchange factor 3 | 1.418525857 | ↑ | 0.01366 |
| O09032 | ELAV-like protein 4 | 1.419253432 | ↑ | 0.003531 |
| P04466 | Myosin regulatory light chain 2, skeletal muscle isoform | 1.420416619 | ↑ | 0.001761 |
| Q05030 | Platelet-derived growth factor receptor beta | 1.420742075 | ↑ | 0.027165 |
| P11506 | Plasma membrane calcium-transporting ATPase 2 | 1.425274197 | ↑ | 0.008943 |
| Q63635 | Syntaxin-6 | 1.425659817 | ↑ | 0.009855 |
| O54701 | Sodium/potassium/calcium exchanger 2 | 1.427302869 | ↑ | 0.006435 |

Continue Table

| Gene IDs | Protein names | Fold change | | *p*-value |
|---|---|---|---|---|
| D4ABB8 | Probable phospholipid-transporting ATPase IIB | 1.427961398 | ↑ | 0.001151 |
| O89040 | 1-phosphatidylinositol 4,5-bisphosphate phosphodiesterase beta-2 | 1.428077337 | ↑ | 0.000493 |
| Q6P7S1 | Acid ceramidase | 1.42863542 | ↑ | 0.013056 |
| Q9QYG8 | Uridine-cytidine kinase 2 | 1.431536973 | ↑ | 0.022352 |
| Q498T9 | Volume-regulated anion channel subunit LRRC8C | 1.435991117 | ↑ | 0.026201 |
| Q9JM47 | Calsenilin | 1.436338957 | ↑ | 0.00793 |
| Q9JID2 | Guanine nucleotide-binding protein subunit alpha-11 | 1.438301582 | ↑ | 0.009553 |
| Q9QYP2 | Cadherin EGF LAG seven-pass G-type receptor 2 (Fragment) | 1.438979349 | ↑ | 0.00735 |
| P11345 | RAF proto-oncogene serine/threonine-protein kinase | 1.440932761 | ↑ | 0.020181 |
| Q63639 | Retinal dehydrogenase 2 | 1.441702005 | ↑ | 0.005774 |
| P70550 | Ras-related protein Rab-8B | 1.441816132 | ↑ | 0.012544 |
| Q505J6 | Mitochondrial glutamate carrier 2 | 1.441844484 | ↑ | 0.008943 |
| P56819 | Beta-secretase 1 | 1.442144801 | ↑ | 0.000421 |
| P51111 | Huntingtin | 1.442372541 | ↑ | 0.00718 |
| Q5FVN8 | WD repeat, SAM and U-box domain-containing protein 1 | 1.442962171 | ↑ | 0.021886 |
| Q3B7T6 | CTD nuclear envelope phosphatase 1 | 1.443014761 | ↑ | 0.046127 |
| P55260 | Annexin A4 | 1.443578618 | ↑ | 0.001634 |
| Q8CGS4 | Charged multivesicular body protein 3 | 1.443797851 | ↑ | 0.034233 |
| P41498 | Low molecular weight phosphotyrosine protein phosphatase | 1.446956839 | ↑ | 0.032995 |
| Q5XIG6 | N-acetylgalactosamine kinase | 1.448070216 | ↑ | 0.005194 |
| Q9WVA1 | Mitochondrial import inner membrane translocase subunit Tim8 A | 1.453370382 | ↑ | 0.002764 |
| Q7TPB1 | T-complex protein 1 subunit delta | 1.455319196 | ↑ | 0.030513 |
| Q6DKG0 | N-alpha-acetyltransferase 35, NatC auxiliary subunit | 1.45534942 | ↑ | 0.004332 |
| Q6Q0N0 | Calsyntenin-1 | 1.456142864 | ↑ | 0.008148 |

Continue Table

| Gene IDs | Protein names | Fold change | | *p*-value |
|---|---|---|---|---|
| Q64591 | 2,4-dienoyl-CoA reductase, mitochondrial | 1.456388031 | ↑ | 0.025646 |
| P49791 | Nuclear pore complex protein Nup153 | 1.45735447 | ↑ | 0.033791 |
| Q5PPG6 | Nucleosome assembly protein 1-like 5 | 1.458061164 | ↑ | 0.004268 |
| P68403 | Protein kinase C beta type | 1.461098637 | ↑ | 0.00589 |
| Q5XII0 | Mammalian ependymin-related protein 1 | 1.464245186 | ↑ | 0.029884 |
| Q5FWT1 | Protein FAM98A | 1.46667752 | ↑ | 0.004608 |
| P18645 | UDP-glucose 4-epimerase | 1.466742337 | ↑ | 0.039246 |
| Q5U2U0 | ATP-dependent Clp protease ATP-binding subunit clpX-like, mitochondrial | 1.467044513 | ↑ | 0.033318 |
| P52555 | Endoplasmic reticulum resident protein 29 | 1.467411463 | ↑ | 0.036866 |
| D3Z9R8 | ATP synthase subunit ATP5MPL, mitochondrial | 1.468177249 | ↑ | 0.00197 |
| O88453 | Scaffold attachment factor B1 | 1.468687312 | ↑ | 0.045687 |
| P60203 | Myelin proteolipid protein | 1.469142211 | ↑ | 0.012537 |
| Q9EPZ7 | Solute carrier organic anion transporter family member 1C1 | 1.469569735 | ↑ | 0.004325 |
| Q66H40 | High mobility group nucleosome-binding domain-containing protein 3 | 1.471055182 | ↑ | 0.018021 |
| Q711G3 | Isoamyl acetate-hydrolyzing esterase 1 homolog | 1.473183446 | ↑ | 0.007303 |
| Q9QXU9 | ProSAAS | 1.473206852 | ↑ | 0.029722 |
| P97878 | Exocyst complex component 5 | 1.474606551 | ↑ | 0.049097 |
| Q68FU1 | Pleckstrin homology domain-containing family F member 1 | 1.476282374 | ↑ | 0.032721 |
| Q9Z244 | GMP reductase 1 | 1.477479717 | ↑ | 0.004781 |
| P0CE43 | Growth factor receptor-bound protein 10 | 1.479515411 | ↑ | 0.01014 |
| Q64303 | Serine/threonine-protein kinase PAK 2 | 1.480314424 | ↑ | 0.023828 |
| P63281 | SUMO=-conjugating enzyme UBC9 | 1.483483355 | ↑ | 0.001588 |
| Q9JKA9 | Potassium/sodium hyperpolarization-activated cyclic nucleotide-gated channel 2 | 1.486091274 | ↑ | 0.005747 |
| Q8VHW5 | Voltage-dependent calcium channel gamma-8 subunit | 1.488971499 | ↑ | 0.007399 |

Continue Table

| Gene IDs | Protein names | Fold change | | p-value |
|---|---|---|---|---|
| P12839 | Neurofilament medium polypeptide | 1.492764696 | ↑ | 0.045562 |
| P02683 | Neuronal vesicle trafficking-associated protein 1 | 1.498906237 | ↑ | 0.034195 |
| P04355 | Metallothionein-2 | 1.501094945 | ↑ | 0.030025 |
| P22462 | Potassium voltage-gated channel subfamily C member 2 | 1.502915862 | ↑ | 0.018999 |
| P29419 | ATP synthase subunit e, mitochondrial | 1.505324925 | ↑ | 0.001567 |
| P68101 | Eukaryotic translation initiation factor 2 subunit 1 | 1.505406241 | ↑ | 0.045691 |
| Q9ESV1 | Leucine zipper protein 1 | 1.506712348 | ↑ | 0.011445 |
| Q63481 | Ras-related protein Rab-7L1 | 1.507570152 | ↑ | 0.008404 |
| P61808 | Stannin | 1.509133785 | ↑ | 5.7E-05 |
| Q91ZQ0 | Vacuole membrane protein 1 | 1.509659213 | ↑ | 0.01753 |
| P55770 | NHP2-like protein 1 | 1.511011349 | ↑ | 0.020206 |
| P04041 | Glutathione peroxidase 1 | 1.512616001 | ↑ | 0.005669 |
| P70600 | Protein-tyrosine kinase 2-beta | 1.513444596 | ↑ | 0.043643 |
| Q71QF9 | Prickle-like protein 1 | 1.51398318 | ↑ | 0.025575 |
| O54852 | Potassium voltage-gated channel subfamily H member 7 | 1.516089441 | ↑ | 0.021003 |
| Q63358 | Unconventional myosin-IXb | 1.516161915 | ↑ | 0.007724 |
| Q5U1Y4 | 1,5-anhydro-D-fructose reductase | 1.51647673 | ↑ | 0.016221 |
| P49801 | Regulator of G-protein signaling 6 (Fragment) | 1.517296313 | ↑ | 0.004843 |
| Q07014 | Tyrosine-protein kinase Lyn | 1.51893413 | ↑ | 0.000114 |
| P02401 | 60S acidic ribosomal protein P2 | 1.520755393 | ↑ | 0.006294 |
| D4ABB4 | F-box/LRR-repeat protein 15 | 1.521405269 | ↑ | 0.001766 |
| Q99PM1 | TOX high mobility group box family member 4 | 1.523799994 | ↑ | 0.011912 |
| Q5I0K3 | Citramalyl-CoA lyase, mitochondrial | 1.527652331 | ↑ | 0.044828 |
| Q8K3P6 | Calcium-binding mitochondrial carrier protein SCaMC-2 | 1.528785533 | ↑ | 0.027405 |

Continue Table

| Gene IDs | Protein names | Fold change | | p-value |
|---|---|---|---|---|
| Q5BK81 | Prostaglandin reductase 2 | 1.531813594 | ↑ | 0.009412 |
| D4AD37 | Golgi-resident adenosine 3',5'-bisphosphate 3'-phosphatase | 1.534510421 | ↑ | 0.001683 |
| Q5GH59 | XK-related protein 4 | 1.535415535 | ↑ | 0.011172 |
| D4A3U0 | G-protein coupled receptor 22 | 1.535988803 | ↑ | 0.010828 |
| Q8CGU6 | Nicastrin | 1.5456779 | ↑ | 0.000526 |
| P84083 | ADP-ribosylation factor 5 | 1.547547183 | ↑ | 0.023433 |
| P15387 | Potassium voltage-gated channel subfamily B member 1 | 1.549177076 | ↑ | 0.001121 |
| G3V9R8 | Heterogeneous nuclear ribonucleoprotein C | 1.549309424 | ↑ | 0.013087 |
| Q62645 | Glutamate receptor ionotropic, NMDA 2D | 1.551915368 | ↑ | 0.000762 |
| Q812E4 | Synaptotagmin-like protein 5 | 1.55450058 | ↑ | 0.040429 |
| P86091 | Transmembrane protease serine 7 | 1.555112259 | ↑ | 0.005338 |
| Q6P7A9 | Lysosomal alpha-glucosidase | 1.555453532 | ↑ | 0.028965 |
| Q66X93 | Staphylococcal nuclease domain-containing protein 1 | 1.561777148 | ↑ | 0.003932 |
| P29147 | D-beta-hydroxybutyrate dehydrogenase, mitochondrial | 1.563943456 | ↑ | 0.007651 |
| Q5XIA8 | Growth hormone-inducible transmembrane protein | 1.565503284 | ↑ | 0.007507 |
| P70567 | Tropomodulin-1 | 1.56592867 | ↑ | 0.006087 |
| O88457 | Sodium channel protein type 11 subunit alpha | 1.566935437 | ↑ | 0.025382 |
| Q63945 | Protein SET | 1.567207141 | ↑ | 0.008874 |
| Q91Z80 | Liprin-alpha-4 (Fragment) | 1.568755889 | ↑ | 0.004105 |
| P35434 | ATP synthase subunit delta, mitochondrial | 1.569823233 | ↑ | 0.004676 |
| Q62667 | Major vault protein | 1.570144071 | ↑ | 0.021251 |
| D4AAT7 | ATP-dependent (S)-NAD(P)H-hydrate dehydratase | 1.572409696 | ↑ | 0.017943 |
| Q5I0I4 | Distal membrane-arm assembly complex protein 2 | 1.572423024 | ↑ | 0.039105 |
| P0C8E4 | Mitogen-activated protein kinase kinase kinase 7 | 1.57378027 | ↑ | 0.040823 |

Continue Table

| Gene IDs | Protein names | Fold change | | *p*-value |
|---|---|---|---|---|
| Q9HB97 | Alpha-parvin | 1.576913216 | ↑ | 0.00802 |
| P20717 | Protein-arginine deiminase type-2 | 1.578329979 | ↑ | 0.030997 |
| P61805 | Dolichyl-diphosphooligosaccharide--protein glycosyltransferase subunit DAD1 | 1.578418694 | ↑ | 0.001143 |
| P15651 | Short-chain specific acyl-CoA dehydrogenase, mitochondrial | 1.579638433 | ↑ | 0.009044 |
| Q5K2P9 | Polyserase-2 | 1.58094241 | ↑ | 0.035777 |
| O54888 | DNA-directed RNA polymerase I subunit RPA2 | 1.585005026 | ↑ | 0.011704 |
| Q4V7D3 | Macoilin | 1.585617542 | ↑ | 0.048338 |
| Q9WU49 | Calcium-regulated heat stable protein 1 | 1.586212821 | ↑ | 0.002051 |
| Q5U2S0 | Arginine/serine-rich protein 1 | 1.590029017 | ↑ | 0.003922 |
| Q6TXG9 | Swi5-dependent recombination DNA repair protein 1 homolog | 1.593207269 | ↑ | 0.006988 |
| O35147 | Bcl2-associated agonist of cell death | 1.595222251 | ↑ | 0.0024 |
| P40241 | CD9 antigen | 1.596275974 | ↑ | 0.025827 |
| Q07984 | Translocon-associated protein subunit delta | 1.597065556 | ↑ | 0.01335 |
| Q04931 | FACT complex subunit SSRP1 | 1.601108073 | ↑ | 0.02755 |
| P15865 | Histone H1.4 | 1.601445185 | ↑ | 0.044036 |
| D3ZEF4 | Cullin-7 | 1.60431138 | ↑ | 0.013841 |
| P37727 | Rab proteins geranylgeranyltransferase component A 1 | 1.605945619 | ↑ | 0.017184 |
| P07151 | Beta-2-microglobulin | 1.607411366 | ↑ | 0.017331 |
| Q32PZ3 | Protein unc-45 homolog A | 1.609739444 | ↑ | 0.027838 |
| O70352 | CD82 antigen | 1.610516046 | ↑ | 0.002195 |
| P08485 | Muscarinic acetylcholine receptor M4 | 1.611643002 | ↑ | 0.000269 |
| Q4V8B7 | Inactive hydroxysteroid dehydrogenase-like protein 1 | 1.611684672 | ↑ | 0.021517 |
| Q7TMD5 | Zinc finger CCCH domain-containing protein 14 | 1.611793744 | ↑ | 0.012736 |
| D3ZLH5 | Plexin-B3 | 1.612165101 | ↑ | 0.000768 |

Continue Table

| Gene IDs | Protein names | Fold change | | p-value |
|---|---|---|---|---|
| O09175 | Aminopeptidase B | 1.613994236 | ↑ | 0.006148 |
| B0BN18 | Prefoldin subunit 2 | 1.616986048 | ↑ | 0.008829 |
| Q9EPH2 | MARCKS-related protein | 1.618173325 | ↑ | 0.005634 |
| P16884 | Neurofilament heavy polypeptide | 1.618801384 | ↑ | 0.005634 |
| B2RYU6 | Trafficking protein particle complex subunit 2-like protein | 1.619470891 | ↑ | 0.024028 |
| Q63448 | Peroxisomal acyl-coenzyme A oxidase 3 | 1.620456092 | ↑ | 0.018588 |
| Q8CH84 | ELAV-like protein 2 | 1.628322082 | ↑ | 0.001375 |
| Q5XI46 | ATPase PAAT | 1.630393012 | ↑ | 0.000706 |
| B2DD29 | Serine/threonine-protein kinase BRSK1 | 1.632030992 | ↑ | 0.002964 |
| O08679 | Serine/threonine-protein kinase MARK2 | 1.632827854 | ↑ | 0.007336 |
| P63138 | Gamma-aminobutyric acid receptor subunit beta-2 | 1.634079661 | ↑ | 0.033144 |
| P05942 | Protein S100-A4 | 1.635277818 | ↑ | 0.021886 |
| Q700K0 | SCO=-spondin | 1.637328159 | ↑ | 0.033245 |
| Q9EPA0 | Dystrophin-related protein 2 | 1.638628903 | ↑ | 0.008179 |
| Q62764 | Y-box-binding protein 3 | 1.645902248 | ↑ | 0.03889 |
| Q9QYJ4 | ATP-binding cassette sub-family B member 9 | 1.647230063 | ↑ | 0.038964 |
| Q5XI28 | Ribonucleoprotein PTB-binding 1 | 1.650028865 | ↑ | 0.016654 |
| P52188 | ATP-sensitive inward rectifier potassium channel 12 | 1.652454923 | ↑ | 0.035621 |
| Q9R1R4 | Tudor domain-containing protein 7 | 1.654338939 | ↑ | 0.03671 |
| Q3B8N7 | TSC22 domain family protein 4 | 1.658229353 | ↑ | 0.002555 |
| Q6MGD0 | Protein CutA | 1.66436305 | ↑ | 0.004185 |
| Q66HA6 | ADP-ribosylation factor-like protein 8B | 1.665702135 | ↑ | 0.007081 |
| Q6MG82 | Proline-rich transmembrane protein 1 | 1.684394579 | ↑ | 0.001376 |
| Q7TQ82 | 28S ribosomal protein S10, mitochondrial | 1.685824059 | ↑ | 0.000732 |

Continue Table

| Gene IDs | Protein names | Fold change | | p-value |
|---|---|---|---|---|
| Q6AYQ4 | Transmembrane protein 109 | 1.688726847 | ↑ | 0.038812 |
| Q6P0K8 | Junction plakoglobin | 1.689782323 | ↑ | 0.020315 |
| Q9JIL8 | DNA repair protein RAD50 | 1.691089595 | ↑ | 0.010915 |
| P42533 | Tissue factor | 1.691117238 | ↑ | 0.010298 |
| B1H267 | Sorting nexin-5 | 1.702688835 | ↑ | 0.007255 |
| O89046 | Coronin-1B | 1.704258409 | ↑ | 0.026571 |
| Q03410 | Synaptonemal complex protein 1 | 1.707866648 | ↑ | 0.005226 |
| O08984 | Delta(14)-sterol reductase LBR | 1.708492669 | ↑ | 0.002613 |
| C0HLM6 | Small integral membrane protein 20 | 1.710619957 | ↑ | 0.016686 |
| P80299 | Bifunctional epoxide hydrolase 2 | 1.714877241 | ↑ | 0.000522 |
| P0DMW1 | Heat shock 70 kDa protein 1B | 1.718002147 | ↑ | 0.010184 |
| P0DMW0 | Heat shock 70 kDa protein 1A | 1.718002147 | ↑ | 0.010183 |
| Q62991 | Sec1 family domain-containing protein 1 | 1.731494541 | ↑ | 0.020016 |
| P29101 | Synaptotagmin-2 | 1.736585813 | ↑ | 0.004474 |
| P70645 | Bleomycin hydrolase | 1.738552496 | ↑ | 0.001843 |
| Q68G30 | Kinesin light chain 3 | 1.739043611 | ↑ | 0.001159 |
| P10715 | Cytochrome c, testis-specific | 1.739828462 | ↑ | 0.024518 |
| P08082 | Clathrin light chain B | 1.744348628 | ↑ | 0.017583 |
| B0BND0 | Glycerophosphocholine cholinephosphodiesterase ENPP6 | 1.745375239 | ↑ | 0.000604 |
| Q01205 | Dihydrolipoyllysine-residue succinyltransferase component of 2-oxoglutarate dehydrogenase complex, mitochondrial | 1.747919782 | ↑ | 0.032873 |
| D3ZLZ7 | Inosine-5'-monophosphate dehydrogenase 1 | 1.751123757 | ↑ | 9.51E-05 |
| P00762 | Anionic trypsin-1 | 1.751540667 | ↑ | 0.001995 |
| Q1HCL7 | NAD kinase 2, mitochondrial | 1.75203939 | ↑ | 0.007014 |

Continue Table

| Gene IDs | Protein names | Fold change | | p-value |
|---|---|---|---|---|
| P70610 | Double C2-like domain-containing protein beta | 1.756675072 | ↑ | 0.024685 |
| P08426 | Cationic trypsin-3 | 1.76224241 | ↑ | 0.004975 |
| O35921 | Excitatory amino acid transporter 4 | 1.768606986 | ↑ | 0.016723 |
| G3V9D0 | Protein O-glucosyltransferase 1 | 1.76933902 | ↑ | 0.000143 |
| Q68FU7 | Ubiquinone biosynthesis monooxygenase COQ6, mitochondrial | 1.771534576 | ↑ | 0.039401 |
| Q4V887 | Zinc transporter ZIP6 | 1.791937365 | ↑ | 0.047196 |
| Q642C0 | DnaJ homolog subfamily C member 8 | 1.793043549 | ↑ | 0.015681 |
| Q9R1T5 | Aspartoacylase | 1.802537496 | ↑ | 0.044639 |
| Q9JKD6 | Claudin-5 | 1.803477638 | ↑ | 0.004186 |
| P52303 | AP-1 complex subunit beta-1 | 1.803759063 | ↑ | 0.000474 |
| O70199 | UDP-glucose 6-dehydrogenase | 1.808276572 | ↑ | 0.002208 |
| P0C0A9 | Small VCP/p97-interacting protein | 1.811452921 | ↑ | 0.018096 |
| Q63530 | Phosphotriesterase-related protein | 1.811571408 | ↑ | 0.016072 |
| Q9EPX4 | P2Y purinoceptor 12 | 1.811602846 | ↑ | 0.017407 |
| Q63312 | Pleckstrin homology-like domain family B member 1 (Fragment) | 1.815859867 | ↑ | 0.013548 |
| Q08326 | Guanine nucleotide exchange factor MSS4 | 1.822421357 | ↑ | 0.034441 |
| Q4FZU6 | Annexin A8 | 1.823805559 | ↑ | 0.034441 |
| P12369 | cAMP-dependent protein kinase type II-beta regulatory subunit | 1.82849054 | ↑ | 0.04158 |
| Q4G008 | Uncharacterized protein KIAA0930 homolog | 1.830892257 | ↑ | 0.010564 |
| P02803 | Metallothionein-1 | 1.831963541 | ↑ | 0.000451 |
| B2RYF7 | WASH complex subunit 1 | 1.834241192 | ↑ | 0.011007 |
| P35284 | Ras-related protein Rab-12 | 1.837394794 | ↑ | 0.009788 |
| Q5RJQ4 | NAD-dependent protein deacetylase sirtuin-2 | 1.843677109 | ↑ | 0.018 |
| A1L1J9 | Lipase maturation factor 2 | 1.845275291 | ↑ | 0.049339 |

Continue Table

| Gene IDs | Protein names | Fold change | | p-value |
|---|---|---|---|---|
| P47198 | 60S ribosomal protein L22 | 1.854441466 | ↑ | 0.03469 |
| O88801 | Homer protein homolog 2 | 1.864792844 | ↑ | 0.002026 |
| D3ZWJ9 | Protein FAM234B | 1.868968961 | ↑ | 0.000329 |
| P58821 | Neuron-specific vesicular protein calcyon | 1.870532894 | ↑ | 0.000184 |
| Q5XIC2 | Evolutionarily conserved signaling intermediate in Toll pathway, mitochondrial | 1.876453743 | ↑ | 0.019162 |
| P04937 | Fibronectin | 1.878119294 | ↑ | 0.041227 |
| Q07141 | Transducin-like enhancer protein 4 | 1.890986754 | ↑ | 0.012974 |
| P30427 | Plectin | 1.900652597 | ↑ | 0.045007 |
| P54759 | Ephrin type-A receptor 7 | 1.937377809 | ↑ | 0.042111 |
| Q6AYQ8 | Acylpyruvase FAHD1, mitochondrial | 1.937531923 | ↑ | 0.037636 |
| P18445 | 60S ribosomal protein L27a | 1.94152265 | ↑ | 0.030833 |
| Q792I0 | Protein lin-7 homolog C | 1.954337727 | ↑ | 0.005785 |
| P30553 | Gastrin/cholecystokinin type B receptor | 1.967706226 | ↑ | 0.000969 |
| O35263 | Platelet-activating factor acetylhydrolase IB subunit gamma | 1.969160992 | ↑ | 0.047648 |
| P54290 | Voltage-dependent calcium channel subunit alpha-2/delta-1 | 1.99513784 | ↑ | 0.045385 |
| Q8K3F3 | Protein phosphatase 1 regulatory subunit 14B | 1.99515451 | ↑ | 0.000249 |
| P06302 | Prothymosin alpha | 2.008495451 | ↑ | 0.008665 |
| P49620 | Diacylglycerol kinase gamma | 2.015496142 | ↑ | 0.028455 |
| D4A1F2 | [F-actin]-monooxygenase MICAL2 | 2.029738774 | ↑ | 0.022161 |
| P00786 | Pro-cathepsin H | 2.034270255 | ↑ | 0.001382 |
| P36202 | PDZ and LIM domain protein 4 | 2.040378213 | ↑ | 0.019019 |
| Q63556 | Serine protease inhibitor A3M (Fragment) | 2.040385892 | ↑ | 0.00702 |
| E9PTA2 | Transient receptor potential cation channel subfamily M member 2 | 2.041769999 | ↑ | 0.019729 |
| Q80W57 | Broad substrate specificity ATP-binding cassette transporter ABCG2 | 2.071516224 | ↑ | 0.037139 |

Continue Table

| Gene IDs | Protein names | Fold change | | p-value |
|---|---|---|---|---|
| Q9QYM0 | Multidrug resistance-associated protein 5 | 2.096044281 | ↑ | 0.011813 |
| P15390 | Sodium channel protein type 4 subunit alpha | 2.1052193 | ↑ | 0.003734 |
| P33671 | Syndecan-3 | 2.1072888 | ↑ | 0.034018 |
| O08662 | Phosphatidylinositol 4-kinase alpha | 2.129379086 | ↑ | 0.003051 |
| Q4V888 | Type 2 phosphatidylinositol 4,5-bisphosphate 4-phosphatase | 2.139147847 | ↑ | 0.016758 |
| Q9JJL4 | Rho-related GTP-binding protein RhoQ | 2.176330335 | ↑ | 0.005733 |
| Q63663 | Guanylate-binding protein 1 | 2.248490856 | ↑ | 0.014664 |
| P59649 | FXYD domain-containing ion transport regulator 7 | 2.254659387 | ↑ | 0.022088 |
| P0C588 | Metal transporter CNNM4 | 2.287605345 | ↑ | 0.046547 |
| P63140 | Nuclear transcription factor Y subunit beta | 2.296185916 | ↑ | 0.024296 |
| Q68FP9 | Conserved oligomeric Golgi complex subunit 6 | 2.331202411 | ↑ | 0.00619 |
| Q8CHJ1 | Phosphatidylinositol glycan anchor biosynthesis class U protein | 2.333882762 | ↑ | 0.036138 |
| Q06437 | Pyruvate dehydrogenase E1 component subunit alpha, testis-specific form, mitochondrial | 2.360984478 | ↑ | 0.00333 |
| P04646 | 60S ribosomal protein L35a | 2.366906621 | ↑ | 0.044473 |
| P07150 | Annexin A1 | 2.38342024 | ↑ | 0.012672 |
| P54708 | Potassium-transporting ATPase alpha chain 2 | 2.433972285 | ↑ | 1.18E-05 |
| Q9JK41 | High affinity copper uptake protein 1 | 2.455297827 | ↑ | 0.011005 |
| A0JPP1 | Dr1-associated corepressor | 2.538969923 | ↑ | 0.003914 |
| Q10758 | Keratin, type II cytoskeletal 8 | 2.543029445 | ↑ | 0.003418 |
| Q0VGK3 | Glycerate kinase | 2.564680169 | ↑ | 0.003614 |
| P12939 | Cytochrome P450 2D10 | 2.612225087 | ↑ | 0.040722 |
| P10633 | Cytochrome P450 2D1 | 2.612225087 | ↑ | 0.003804 |
| P22002 | Voltage-dependent L-type calcium channel subunit alpha-1C | 2.713473647 | ↑ | 0.008634 |
| P19132 | Ferritin heavy chain | 2.715158894 | ↑ | 0.024732 |

Continue Table

| Gene IDs | Protein names | Fold change | | *p*-value |
|---|---|---|---|---|
| Q6P742 | Proteolipid protein 2 | 2.720042469 | ↑ | 0.0422 |
| P26376 | Interferon-induced transmembrane protein 3 | 2.732079919 | ↑ | 0.001792 |
| Q5XIA2 | SUZ domain-containing protein 1 | 2.763657671 | ↑ | 0.041204 |
| P16391 | RT1 class I histocompatibility antigen, AA alpha chain | 2.789357332 | ↑ | 0.026837 |
| P15978 | Class I histocompatibility antigen, Non-RT1.A alpha-1 chain | 2.789357332 | ↑ | 0.021886 |
| Q62714 | Neutrophil antibiotic peptide NP-4 | 2.803483663 | ↑ | 0.020014 |
| P28064 | Proteasome subunit beta type-8 | 2.843482902 | ↑ | 0.010768 |
| Q6IG12 | Keratin, type II cytoskeletal 7 | 2.891141428 | ↑ | 0.000454 |
| Q6IFW6 | Keratin, type I cytoskeletal 10 | 2.894194615 | ↑ | 0.039541 |
| P97523 | Hepatocyte growth factor receptor | 2.920824424 | ↑ | 0.007129 |
| Q9EQZ1 | TSC22 domain family protein 3 | 2.948048844 | ↑ | 0.046047 |
| Q6IMF3 | Keratin, type II cytoskeletal 1 | 3.156789006 | ↑ | 0.042303 |
| Q5M887 | Trafficking protein particle complex subunit 13 | 3.18294173 | ↑ | 0.03817 |
| P50116 | Protein S100-A9 | 3.296914794 | ↑ | 0.032277 |
| Q62622 | Eukaryotic translation initiation factor 4E-binding protein 1 | 3.307334727 | ↑ | 0.020443 |
| O88813 | Long-chain-fatty-acid--CoA ligase 5 | 3.312913002 | ↑ | 0.010506 |
| P68136 | Actin, alpha skeletal muscle | 3.366540627 | ↑ | 0.003883 |
| Q63486 | Ras-related GTP-binding protein A | 3.38361832 | ↑ | 0.015699 |
| O70127 | Bile salt export pump | 3.420119426 | ↑ | 0.017414 |
| P63095 | Guanine nucleotide-binding protein G(s) subunit alpha isoforms short | 3.475602966 | ↑ | 0.014647 |
| Q5FVL7 | Protein kintoun | 3.55204641 | ↑ | 0.022391 |
| P83953 | Importin subunit alpha-5 | 3.743465242 | ↑ | 0.018412 |
| P10688 | 1-phosphatidylinositol 4,5-bisphosphate phosphodiesterase delta-1 | 4.098204753 | ↑ | 0.005898 |
| P04550 | Parathymosin | 4.795301667 | ↑ | 0.013521 |

Continue Table

| Gene IDs | Protein names | Fold change | | *p*-value |
|---|---|---|---|---|
| P50115 | Protein S100-A8 | 7.839777461 | ↑ | 0.035275 |
| Q6LED0 | Histone H3.1 | 8.524910425 | ↑ | 0.031312 |
| P84245 | Histone H3.3 | 11.33379317 | ↑ | 0.012725 |
| Q8VID6 | Dual 3',5'-cyclic-AMP and -GMP phosphodiesterase 11A | 18.18902759 | ↑ | 0.00234 |
| Q6P734 | Plasma protease C1 inhibitor | 79.13097294 | ↑ | 0.004674 |
| | **SZS/LPS comparison group** | | | |
| Gene IDs | Protein names | Fold change | | *p*-value |
| Q6P734 | Plasma protease C1 inhibitor | 0.25847788 | ↓ | 0.047402192 |
| O88813 | Long-chain-fatty-acid--CoA ligase 5 | 0.28887722 | ↓ | 0.023078686 |
| Q5M887 | Trafficking protein particle complex subunit 13 | 0.29828549 | ↓ | 0.001665134 |
| Q62784 | Inositol polyphosphate-4-phosphatase type I A | 0.31494658 | ↓ | 0.009452664 |
| O88279 | Slit homolog 1 protein | 0.3631297 | ↓ | 0.023740382 |
| Q68FP9 | Conserved oligomeric Golgi complex subunit 6 | 0.36555665 | ↓ | 0.010150365 |
| Q5FVL7 | Protein kintoun | 0.3714861 | ↓ | 0.025212875 |
| P80299 | Bifunctional epoxide hydrolase 2 | 0.38364276 | ↓ | 5.77419E-05 |
| O88984 | Nuclear RNA export factor 1 | 0.38431801 | ↓ | 0.032485785 |
| Q0VGK3 | Glycerate kinase | 0.39089811 | ↓ | 0.027997414 |
| Q4FZU6 | Annexin A8 | 0.39278985 | ↓ | 0.011554644 |
| Q9WU68 | Pleckstrin homology domain-containing family B member 1 | 0.39556629 | ↓ | 0.000433556 |
| O35094 | Mitochondrial import inner membrane translocase subunit TIM44 | 0.39732605 | ↓ | 0.022067614 |
| Q6IFW6 | Keratin, type I cytoskeletal 10 | 0.42046723 | ↓ | 0.017042675 |
| Q06437 | Pyruvate dehydrogenase E1 component subunit alpha, testis-specific form, mitochondrial | 0.44242807 | ↓ | 0.027386147 |
| P19132 | Ferritin heavy chain | 0.45896581 | ↓ | 0.014871538 |
| P22002 | Voltage-dependent L-type calcium channel subunit alpha-1C | 0.4616353 | ↓ | 0.002633775 |

Continue Table

| Gene IDs | Protein names | Fold change | | *p*-value |
|---|---|---|---|---|
| Q9JJ31 | Cullin-5 | 0.47808048 | ↓ | 0.00474198 |
| Q9JM47 | Calsenilin | 0.49583921 | ↓ | 0.000529384 |
| Q8K3F3 | Protein phosphatase 1 regulatory subunit 14B | 0.50433329 | ↓ | 0.010120462 |
| Q78P75 | Dynein light chain 2, cytoplasmic | 0.50778845 | ↓ | 0.017654001 |
| Q5PR01 | Pancreatic progenitor cell differentiation and proliferation factor | 0.52556974 | ↓ | 0.011807129 |
| Q4V8H8 | EH domain-containing protein 2 | 0.53971929 | ↓ | 0.031780448 |
| Q5M848 | Calcium release-activated calcium channel protein 1 | 0.54077447 | ↓ | 0.009586259 |
| P07756 | Carbamoyl-phosphate synthase [ammonia], mitochondrial | 0.55128569 | ↓ | 0.001317543 |
| D4A1F2 | [F-actin]-monooxygenase MICAL2 | 0.5583797 | ↓ | 0.006598216 |
| P13444 | S-adenosylmethionine synthase isoform type-1 | 0.56166651 | ↓ | 0.016067567 |
| O08587 | Nuclear pore complex protein Nup50 | 0.56498251 | ↓ | 0.004889636 |
| Q8R4U9 | Serine/threonine-protein kinase Sgk2 | 0.56520767 | ↓ | 0.044680276 |
| P40190 | Interleukin-6 receptor subunit beta | 0.56644497 | ↓ | 0.024610882 |
| P97534 | Peptidyl-prolyl cis-trans isomerase FKBP1B | 0.56670474 | ↓ | 0.004876675 |
| P00786 | Pro-cathepsin H | 0.5697091 | ↓ | 0.00751443 |
| Q8K1P8 | Monocarboxylate transporter 8 | 0.57834103 | ↓ | 0.027637847 |
| D3ZWJ9 | Protein FAM234B | 0.5868441 | ↓ | 0.000574415 |
| Q6AYQ8 | Acylpyruvase FAHD1, mitochondrial | 0.59083245 | ↓ | 0.014607925 |
| Q8VHW5 | Voltage-dependent calcium channel gamma-8 subunit | 0.59761339 | ↓ | 0.017042335 |
| Q63481 | Ras-related protein Rab-7L1 | 0.60470242 | ↓ | 0.006860856 |
| Q63556 | Serine protease inhibitor A3M (Fragment) | 0.61318817 | ↓ | 0.015192851 |
| Q62655 | Transcription factor 4 | 0.61478584 | ↓ | 0.034966851 |
| Q3B7T6 | CTD nuclear envelope phosphatase 1 | 0.61478586 | ↓ | 0.00446606 |
| B2GUW6 | Apoptosis-enhancing nuclease | 0.61802845 | ↓ | 0.010297952 |

Continue Table

| Gene IDs | Protein names | Fold change | | p-value |
|---|---|---|---|---|
| Q5BJ92 | Serine/threonine-protein phosphatase 4 catalytic subunit | 0.61966233 | ↓ | 0.032431632 |
| P49620 | Diacylglycerol kinase gamma | 0.62986052 | ↓ | 0.001519087 |
| Q4FZU2 | Keratin, type II cytoskeletal 6A | 0.6330383 | ↓ | 0.026575357 |
| Q68FU1 | Pleckstrin homology domain-containing family F member 1 | 0.63749539 | ↓ | 0.00761316 |
| Q63055 | ADP-ribosylation factor-related protein 1 | 0.63822471 | ↓ | 0.008984032 |
| Q6P756 | Adaptin ear-binding coat-associated protein 2 | 0.63840198 | ↓ | 0.028406024 |
| P12369 | cAMP-dependent protein kinase type II-beta regulatory subunit | 0.65054717 | ↓ | 0.001589474 |
| Q6AYY8 | Acetyl-coenzyme A transporter 1 | 0.652701 | ↓ | 0.008594771 |
| Q66H59 | N-acetylneuraminate lyase | 0.65502174 | ↓ | 0.027114371 |
| Q1HCL7 | NAD kinase 2, mitochondrial | 0.65977166 | ↓ | 0.000516065 |
| Q62862 | Dual specificity mitogen-activated protein kinase kinase 5 | 0.66110196 | ↓ | 0.032744686 |
| Q62991 | Sec1 family domain-containing protein 1 | 0.66232134 | ↓ | 0.017866744 |
| A0JPN6 | Mediator of RNA polymerase II transcription subunit 22 | 0.66282709 | ↓ | 0.00881434 |
| D3ZHR2 | ATP-binding cassette sub-family D member 1 | 0.66354914 | ↓ | 0.012621949 |
| P54759 | Ephrin type-A receptor 7 | 0.66537878 | ↓ | 0.025566704 |
| O08662 | Phosphatidylinositol 4-kinase alpha | 0.6658053 | ↓ | 0.022402965 |
| Q8K3V5 | Fibronectin type III domain-containing protein 5 | 0.67139626 | ↓ | 0.00387438 |
| O08949 | Transcription initiation factor IIA subunit 1 | 0.67203246 | ↓ | 0.007211184 |
| Q6MGD0 | Protein CutA | 0.67752957 | ↓ | 0.032832566 |
| Q63639 | Retinal dehydrogenase 2 | 0.68384017 | ↓ | 0.038239162 |
| Q6AYA5 | Transmembrane protein 106B | 0.68467661 | ↓ | 0.00168117 |
| Q642B9 | Zinc finger protein 18 | 0.68620784 | ↓ | 0.008545147 |
| Q5U2U0 | ATP-dependent Clp protease ATP-binding subunit clpX-like, mitochondrial | 0.68645383 | ↓ | 0.021039575 |
| Q700K0 | SCO=-spondin | 0.68708288 | ↓ | 0.019703045 |

Continue Table

| Gene IDs | Protein names | Fold change | | *p*-value |
|---|---|---|---|---|
| D3ZLZ7 | Inosine-5'-monophosphate dehydrogenase 1 | 0.68718608 | ↓ | 0.036285823 |
| Q8CG07 | ATPase WRNIP1 | 0.68771903 | ↓ | 0.002186393 |
| P02564 | Myosin-7 | 0.6882023 | ↓ | 0.036617454 |
| P0C588 | Metal transporter CNNM4 | 0.69210378 | ↓ | 0.04404511 |
| Q01205 | Dihydrolipoyllysine-residue succinyltransferase component of 2-oxoglutarate dehydrogenase complex, mitochondrial | 0.69642442 | ↓ | 0.001238941 |
| P29410 | Adenylate kinase 2, mitochondrial | 0.69896972 | ↓ | 0.012175943 |
| Q68FU7 | Ubiquinone biosynthesis monooxygenase COQ6, mitochondrial | 0.7004198 | ↓ | 0.020203093 |
| P31722 | Complement C1q subcomponent subunit C | 0.70564158 | ↓ | 0.04620614 |
| P55213 | Caspase-3 | 0.70900266 | ↓ | 0.016350446 |
| Q6TXG9 | Swi5-dependent recombination DNA repair protein 1 homolog | 0.70938629 | ↓ | 0.041038244 |
| Q32PZ3 | Protein unc-45 homolog A | 0.7103814 | ↓ | 0.046461848 |
| B4F7C5 | Leucine-rich repeat transmembrane neuronal protein 4 | 0.71119567 | ↓ | 0.042824873 |
| Q5FVJ6 | Seipin | 0.71362322 | ↓ | 0.030245716 |
| Q5M9F0 | UPF0705 protein C11orf49 homolog | 1.40502397 | ↑ | 0.030981016 |
| P42346 | Serine/threonine-protein kinase mTOR | 1.40826864 | ↑ | 0.005060057 |
| D4A631 | Brefeldin A-inhibited guanine nucleotide-exchange protein 1 | 1.4346382 | ↑ | 0.0259921 |
| Q3B8P0 | Presenilins-associated rhomboid-like protein, mitochondrial | 1.4407705 | ↑ | 0.049944962 |
| Q63149 | Cadherin-4 (Fragment) | 1.44454239 | ↑ | 9.08099E-05 |
| Q6P6S3 | B box and SPRY domain-containing protein | 1.45391009 | ↑ | 0.015294603 |
| P70569 | Unconventional myosin-Vb | 1.46381446 | ↑ | 0.044428219 |
| Q6AXQ5 | 2',5'-phosphodiesterase 12 | 1.4830311 | ↑ | 0.015335545 |
| Q4V7F5 | PIH1 domain-containing protein 1 | 1.496439 | ↑ | 0.010970275 |
| Q9EQH5 | C-terminal-binding protein 2 | 1.51742589 | ↑ | 0.013846748 |

Continue Table

| Gene IDs | Protein names | Fold change | | *p*-value |
|---|---|---|---|---|
| Q66H54 | FTS and Hook-interacting protein | 1.52200531 | ↑ | 0.032060558 |
| P0C1S9 | Diacylglycerol lipase-beta | 1.53387389 | ↑ | 0.005098985 |
| P26769 | Adenylate cyclase type 2 | 1.53816222 | ↑ | 0.021772237 |
| Q498D6 | Fibroblast growth factor receptor 4 | 1.54294392 | ↑ | 0.043449922 |
| Q63186 | Translation initiation factor eIF-2B subunit delta | 1.54475246 | ↑ | 0.022561693 |
| Q6AZ61 | Lysosomal cobalamin transport escort protein LMBD1 | 1.55640844 | ↑ | 0.004092216 |
| Q5BJQ6 | Cleavage stimulation factor subunit 1 | 1.56377004 | ↑ | 0.04147974 |
| Q5FVG2 | Band 4.1-like protein 5 | 1.56631561 | ↑ | 0.033761752 |
| P70581 | Nucleoporin p58/p45 | 1.56803066 | ↑ | 0.011415796 |
| Q5XXR3 | Rho guanine nucleotide exchange factor 6 | 1.60860738 | ↑ | 0.035925546 |
| O55170 | Transcription factor SOX-10 | 1.61198852 | ↑ | 0.032141288 |
| D4A702 | Synaptopodin-2 | 1.62196094 | ↑ | 0.019292554 |
| O35412 | Signal-induced proliferation-associated 1-like protein 1 | 1.62851341 | ↑ | 0.022074242 |
| Q5BJP6 | Ribosome-releasing factor 2, mitochondrial | 1.6340388 | ↑ | 0.000795411 |
| Q9JL55 | Glycerophosphodiester phosphodiesterase 1 | 1.66079055 | ↑ | 0.027488248 |
| Q09426 | 2-hydroxyacylsphingosine 1-beta-galactosyltransferase | 1.6740193 | ↑ | 0.04810594 |
| Q9JHE5 | Sodium-coupled neutral amino acid transporter 2 | 1.69011229 | ↑ | 0.013936947 |
| Q62753 | Syntaxin-binding protein 2 | 1.69481157 | ↑ | 0.029105837 |
| P97738 | Neuronal pentraxin-2 | 1.69852973 | ↑ | 0.010971796 |
| Q99JB3 | 4-galactosyl-N-acetylglucosaminide 3-alpha-L-fucosyltransferase 9 | 1.75067203 | ↑ | 0.010240523 |
| Q5U2X0 | CDKN2A-interacting protein | 1.76624072 | ↑ | 0.007685891 |
| Q9EPI6 | NMDA receptor synaptonuclear signaling and neuronal migration factor | 1.77370393 | ↑ | 0.028890947 |
| Q66H60 | Coiled-coil domain-containing protein 146 | 1.80692307 | ↑ | 0.039781364 |
| Q5RKH6 | Protein OS-9 | 1.81088803 | ↑ | 0.004877529 |

Continue Table

| Gene IDs | Protein names | Fold change | | *p*-value |
|---|---|---|---|---|
| P59824 | Interleukin-1 receptor accessory protein-like 1 | 1.82497274 | ↑ | 0.029526877 |
| P50237 | Sulfotransferase 1C1 | 1.85210523 | ↑ | 0.048933198 |
| P28840 | Neuroendocrine convertase 1 | 1.88603294 | ↑ | 0.040727699 |
| Q9WVR7 | Protein phosphatase 1F | 1.89368895 | ↑ | 0.033656686 |
| Q6PEC3 | Protein YIF1B | 1.89925174 | ↑ | 0.039034524 |
| Q62600 | Nitric oxide synthase, endothelial | 1.94061748 | ↑ | 0.047799788 |
| B4F7E8 | Protein Niban 2 | 1.97376581 | ↑ | 0.003084262 |
| Q5XIE0 | Acidic leucine-rich nuclear phosphoprotein 32 family member E | 2.02780036 | ↑ | 0.015748196 |
| Q52KK4 | H/ACA ribonucleoprotein complex non-core subunit NAF1 | 2.03517969 | ↑ | 0.006426929 |
| P53565 | Homeobox protein cut-like 1 | 2.24990709 | ↑ | 0.000911425 |
| Q58DZ9 | Synapse differentiation-inducing gene protein 1 | 2.43138442 | ↑ | 0.032710533 |
| Q6AY64 | UPF0669 protein C6orf120 homolog | 2.48024333 | ↑ | 0.024231872 |
| Q9R0I6 | E3 ubiquitin-protein ligase XIAP | 2.54142322 | ↑ | 0.0082902 |
| F1LM93 | Tyrosine-protein kinase Yes | 2.60892727 | ↑ | 0.011548391 |
| Q7TQ84 | UAP56-interacting factor | 2.74582723 | ↑ | 0.004602914 |
| Q7M6Z5 | Kinesin-like protein KIF27 | 2.75359087 | ↑ | 0.045743679 |
| Q792Q4 | Cysteine-rich PDZ-binding protein | 2.95121005 | ↑ | 0.006813082 |
| Q2PQA9 | Kinesin-1 heavy chain | 3.18551379 | ↑ | 0.002287652 |
| Q99PD6 | Transforming growth factor beta-1-induced transcript 1 protein | 3.96147448 | ↑ | 0.005711225 |
| Q08849 | Syntaxin-3 | 4.52694073 | ↑ | 0.003965376 |
| Q63258 | Integrin alpha-7 | 4.61397378 | ↑ | 0.043207 |
| Q80W92 | Protein VAC14 homolog | 11.7936103 | ↑ | 0.023679766 |
| Q5M971 | Protein AF1q | 28.1933457 | ↑ | 0.025180031 |

**Supplementary Table 2** UHPLC-HR-MS identification of the constituents in SZS decoction.

| Compound NO. | $T_R$ (min) | Measured mass | Calculated mass | Ion form | Proposed fomula | Error (ppm) | Identification |
|---|---|---|---|---|---|---|---|
| 1 | 5.03 | 370.1125 | 370.1133 | $[M+H]^+$ | $C_{16}H_{20}O_9N$ | -1.99 | 3S-N-glc-3-hydroxy-indoleacetic acid |
| 2 | 5.16 | 370.113 | 370.1133 | $[M+H]^+$ | $C_{16}H_{20}O_9N$ | -0.75 | 3S-N-glc-3-hydroxy-indoleacetic acid |
| 3 | 5.3 | 448.1957 | 448.1966 | $[M+H]^+$ | $C_{23}H_{29}O_8N$ | -1.9 | 6-glc-coclaurine |
| 4 | 5.97 | 314.1749 | 314.1751 | $M^+$ | $C_{19}H_{24}O_3N$ | -0.64 | Magnocurarine |
| 5 | 6.96 | 286.1431 | 286.1438 | $[M+H]^+$ | $C_{17}H_{19}O_3N$ | -2.31 | Coclaurine |
| 6 | 6.99 | 354.1174 | 354.1183 | $[M+H]^+$ | $C_{16}H_{20}O_8N$ | -2.75 | N-glc-indoleaceticacid |
| 7 | 7.42 | 328.1545 | 328.1543 | $[M+H]^+$ | $C_{19}H_{22}O_4N$ | -0.01 | Norisocorydine |
| 8 | 7.47 | 282.1124 | 282.1125 | $[M+H]^+$ | $C_{17}H_{16}O_3N$ | -0.14 | Juzirine |
| 9 | 7.57 | 342.1692 | 342.17 | $M^+$ | $C_{20}H_{24}O_4N$ | -2.32 | Zizyphusine |
| 10 | 7.76 | 354.118 | 354.1183 | $[M+H]^+$ | $C_{16}H_{20}O_8N$ | -0.94 | N-glc-indoleaceticacid isomer |
| 11 | 8.62 | 314.1748 | 314.1751 | $M^+$ | $C_{19}H_{24}O_3N$ | -0.86 | Lotusine |
| 12 | 8.98 | 328.1541 | 328.1543 | $[M+H]^+$ | $C_{19}H_{22}O_4N$ | -0.87 | Norisocorydine isomer |
| 13 | 9.24 | 771.2318 | 771.2342 | $[M+H]^+$ | $C_{34}H_{43}O_{20}$ | -3.13 | Unknown |
| 14 | 9.73 | 268.1328 | 268.1332 | $[M+H]^+$ | $C_{17}H_{18}O_2N$ | -1.7 | Caaverine |
| 15 | 10.2 | 595.1653 | 595.1658 | $[M+H]^+$ | $C_{27}H_{31}O_{15}$ | -0.68 | Saponarin |
| 16 | 11.7 | 921.2624 | 921.2659 | $[M+H]^+$ | $C_{42}H_{49}O_{23}$ | -3.83 | 6'''-(4'''-O-glc)-vanilloylspinosin |
| 17 | 11.99 | 891.2519 | 891.2553 | $[M+H]^+$ | $C_{41}H_{47}O_{22}$ | -3.88 | 6'''-(4'''-O-glc)-p-Hydroxybenzoyl |
| 18 | 12.2 | 595.1648 | 595.1658 | $[M+H]^+$ | $C_{27}H_{31}O_{15}$ | -1.61 | Meloside A |
| 19 | 12.51 | 609.1802 | 609.1814 | $[M+H]^+$ | $C_{28}H_{33}O_{15}$ | -1.9 | Isospinosin |
| 20 | 12.73 | 609.1805 | 609.1814 | $[M+H]^+$ | $C_{28}H_{33}O_{15}$ | -1.41 | Spinosin* |
| 21 | 13.12 | 714.2 | 714.2029 | $[M+H]^+$ | $C_{34}H_{36}O_{16}N$ | -3.99 | 6'''-Pyridyloylspinosin |
| 22 | 13.27 | 296.164 | 296.1645 | $[M+H]^+$ | $C_{19}H_{22}O_2N$ | -1.61 | Nuciferine |
| 23 | 13.33 | 195.065 | 195.0652 | $[M+H]^+$ | $C_{10}H_{11}O_4$ | -1.16 | Ferulic acid |
| 24 | 13.58 | 447.1287 | 447.1286 | $[M+H]^+$ | $C_{22}H_{23}O_{10}$ | 0.26 | Swertisin |
| 25 | 14.05 | 759.2104 | 759.2131 | $[M+H]^+$ | $C_{36}H_{39}O_{18}$ | -3.55 | Unknown |
| 26 | 14.4 | 729.2001 | 729.2025 | $[M+H]^+$ | $C_{35}H_{37}O_{17}$ | -3.29 | 6'''hydroxylbenoylspinosin |
| 27 | 14.59 | 709.2312 | 709.2338 | $[M+H]^+$ | $C_{33}H_{41}O_{17}$ | -3.7 | Unknown |
| 28 | 14.87 | 873.3137 | 873.3176 | $[M+H]^+$ | $C_{43}H_{53}O_{19}$ | -4.39 | Unknown |
| 29 | 15.01 | 815.2355 | 815.2393 | $[M+H]^+$ | $C_{39}H_{43}O_{19}$ | -4.6 | 6'''-sinapoylspinosin |
| 30 | 15.4 | 755.215 | 755.2182 | $[M+H]^+$ | $C_{37}H_{39}O_{17}$ | -4.25 | 6'''-Coumaloylspinosin |
| 31 | 15.5 | 785.226 | 785.2287 | $[M+H]^+$ | $C_{38}H_{41}O_{18}$ | -3.48 | 6'''-Feruloylspinosin |
| 32 | 17.5 | 829.2523 | 829.255 | $[M+H]^+$ | $C_{40}H_{45}O_{19}$ | -3.25 | Unknown |
| 33 | 19.64 | 1045.557 | 1045.558 | $[M+H]^+$ | $C_{52}H_{85}O_{21}$ | -1.34 | Jujuboside B |

**Supplementary Table 3** UHPLC-HR-MS identification of SZS compound in rat plasma after oral administration of SZS.

| Compound No. | $T_R$ (min) | Measured mass | Calculated mass | Ion form | Proposed formula | Error (ppm) | Identification |
|---|---|---|---|---|---|---|---|
| 1 | 1.44 | 462.1747 | 462.1759 | $[M+H]^+$ | $C_{23}H_{28}O_9N$ | -2.61 | Coclaurine-glucuronide |
| 2 | 5.05 | 462.1752 | 462.1759 | $[M+H]^+$ | $C_{23}H_{28}O_9N$ | -1.36 | Coclaurine-glucuronide |
| 3 | 6.05 | 462.1749 | 462.1759 | $[M+H]^+$ | $C_{23}H_{28}O_9N$ | -2.01 | Coclaurine-glucuronide |
| 4 | 7.10 | 286.1437 | 286.1438 | $[M+H]^+$ | $C_{17}H_{20}O_3N$ | -0.32 | Coclaurine |
| 5 | 7.67 | 342.1697 | 342.1697 | $M^+$ | $C_{20}H_{24}O_4N$ | -0.98 | Zizyphusine |
| 6 | 7.85 | 342.1702 | 342.1697 | $M^+$ | $C_{20}H_{24}O_4N$ | 0.54 | Zizyphusine isomer |
| 7 | 10.21 | 595.166 | 595.1658 | $[M+H]^+$ | $C_{27}H_{31}O_{15}$ | 0.46 | Saponarin |
| 8 | 10.90 | 309.1228 | 309.1324 | $[M+H]^+$ | $C_{18}H_{17}O_3N_2$ | -1.84 | Unknown |
| 9 | 12.20 | 595.1639 | 595.1658 | $[M+H]^+$ | $C_{27}H_{31}O_{15}$ | -3.2 | Saponarin isomer |
| 10 | 12.75 | 609.1804 | 609.1814 | $[M+H]^+$ | $C_{28}H_{33}O_{15}$ | -2.31 | Spinosin |
| 11 | 13.55 | 447.1282 | 447.1286 | $[M+H]^+$ | $C_{22}H_{23}O_{10}$ | -0.77 | Swertisin |
| 12 | 15.40 | 785.2259 | 785.2287 | $[M+H]^+$ | $C_{38}H_{41}O_{18}$ | -3.63 | 6'''-Feruloylspinosin |

**Supplementary Table 4** Docking energy (Kcal/mol) between potential target proteins and in vivo SZS components.

| Num | Gene Name | Protein ID | **Docking energy (Kcal/mol)** | | | | | | | | |
|---|---|---|---|---|---|---|---|---|---|---|---|
| | | | 6'''-Feruloylspinosin | Swertisin | Spinosin | Nuciferine | Ferulic acid | Isocorydine | Coclaurine | Zizyphusin | Saponarin |
| 1 | ZO-1 | A0A0G2K2P5 | -8.62 | -8.02 | -9.11 | -8.17 | -6.28 | -8.23 | -7.12 | -7.4 | -8 |
| 2 | Occludin | Q6P6T5 | -6.72 | -6.14 | -5.89 | -5.54 | -4.91 | -5.29 | -5.63 | -5.44 | -6.32 |
| 3 | Claudin-5 | Q9JKD6 | -7.31 | -7.62 | -7.63 | -6.37 | -5.24 | -6.34 | -6.58 | -6.91 | -7.26 |
| 4 | E-cadherin | Q9R0T4 | -9.2 | -8.65 | -9.78 | -6.93 | -5.63 | -6.66 | -8.07 | -8.31 | -7.89 |
| 5 | β-catenin | Q9WU82 | -7.17 | -5.45 | -7.89 | -6.68 | -4.67 | -5.93 | -6.52 | -6.83 | -5.68 |
| 6 | FAK1 | O35346 | -10.53 | -9.04 | -8.24 | -8.43 | -6.13 | -7.31 | -7.92 | -7.98 | -7.33 |
| 7 | Dock1 | D3ZZW1 | -9.93 | -8.87 | -8.49 | -7.73 | -6.47 | -7.58 | -8.03 | -7.91 | 7.84 |
| 8 | Rac1 | Q6RUV5 | -9.2 | -6.33 | -6.2 | -7.86 | -4.98 | -7.82 | -7.82 | -5.97 | -6.18 |
| 9 | Wave2 | E9PTF9 | -7.02 | -6.64 | -8.17 | -6.09 | -4.89 | -5.73 | -6.21 | -6.2 | -6.41 |
| 10 | Arp3 | Q4V7C7 | -9.72 | -8.92 | -7.62 | -8.66 | -6.01 | -8.7 | -8.4 | -8.81 | -7.23 |

Note: the chemical structures of SZS components were presented below.

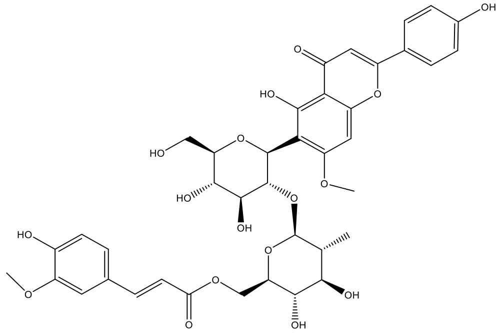


6'''-Feruloylspinosin (FSP)

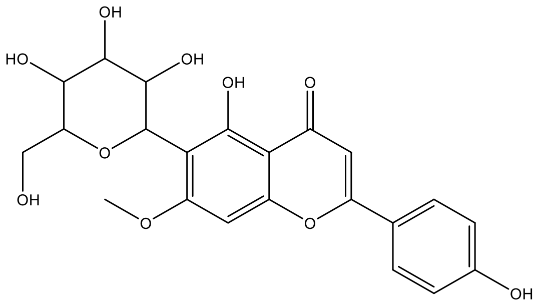


Swertisin (SWE)

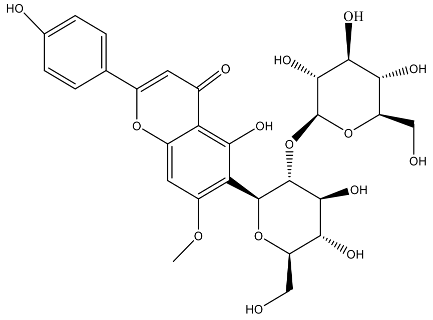


Spinosin (SPI)

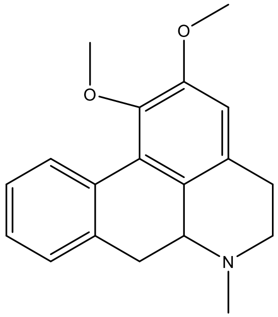

Nuciferine (NUC)

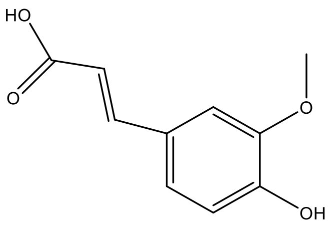

Ferulic acid (FA)

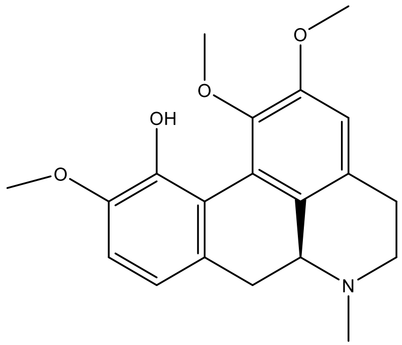

Isocorydine (ICD)

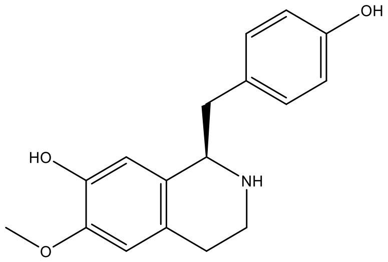

Coclaurine (COC)

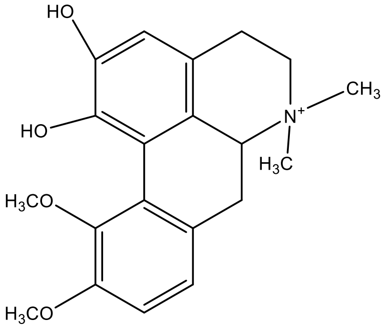

Zizyphusin (ZIZ)

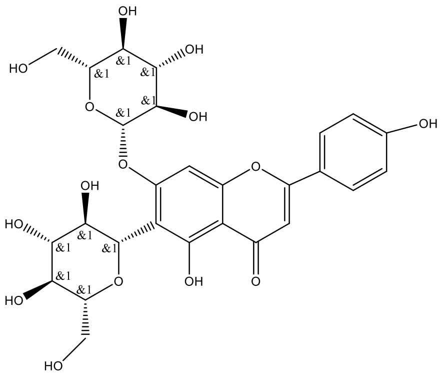

Saponarin (SAP)
