# Supplementary material for: Semen Ziziphi Spinosae attenuates blood–brain barrier dysfunction induced by lipopolysaccharide by targeting the FAK-DOCK180-Rac1-WAVE2-Arp3 signaling pathway
Source: NPJ Sci Food. 2022 Jun 2;6:27. doi: 10.1038/s41538-022-00142-6 (PMC9163036; doi:10.1038/s41538-022-00142-6)
Supplement: Supplementary file 2 — Full scan images [file 41538_2022_142_MOESM2_ESM.pdf]

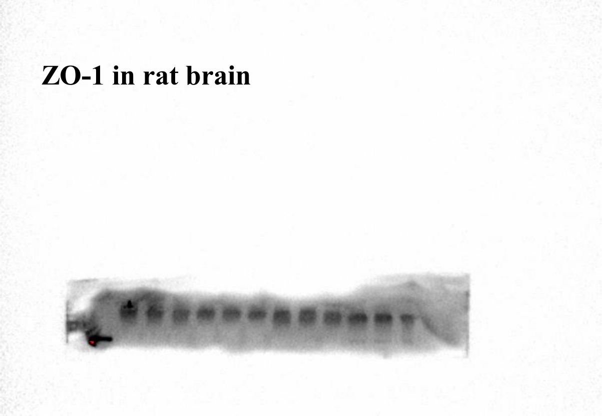
ZO-1 in rat brain

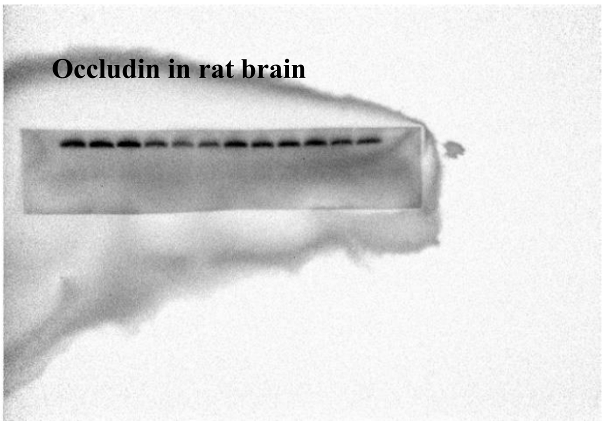
Occludin in rat brain

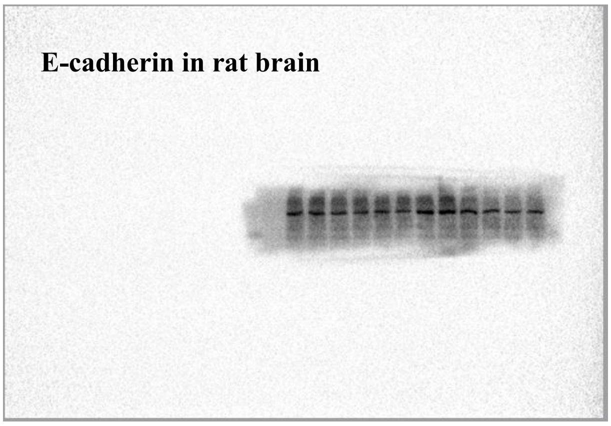
E-cadherin in rat brain

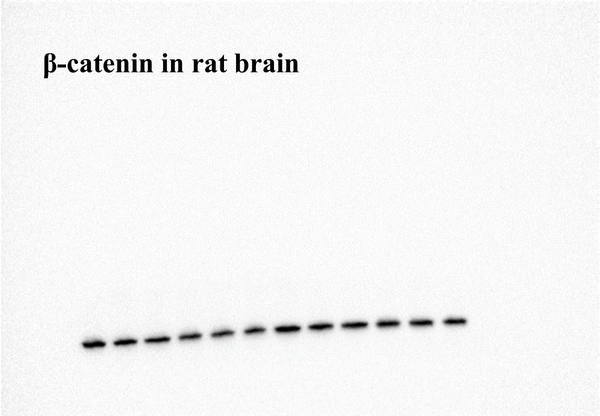
β-catenin in rat brain

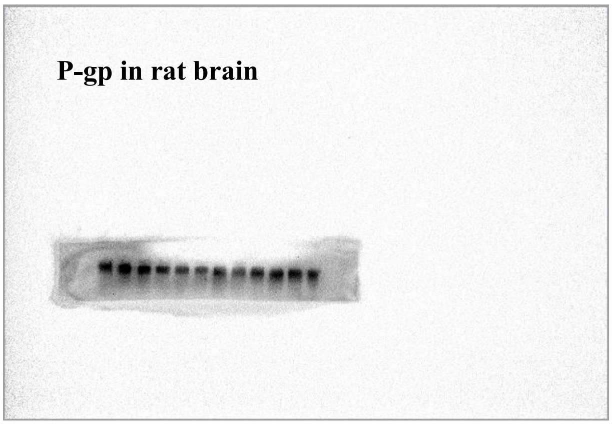
P-gp in rat brain

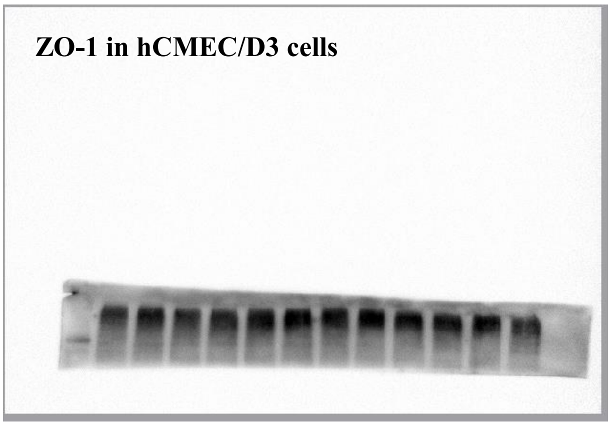
ZO-1 in hCMEC/D3 cells

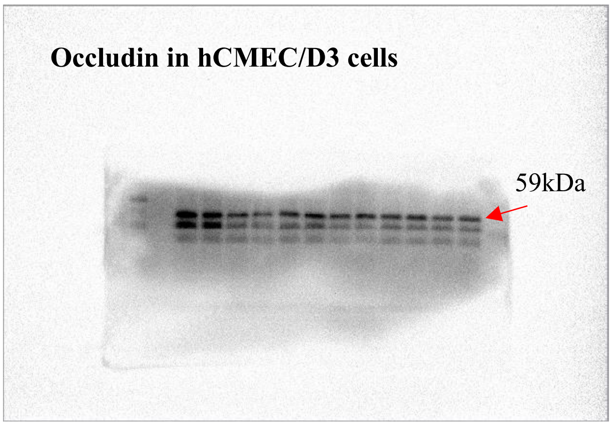
Occludin in hCMEC/D3 cells
59kDa

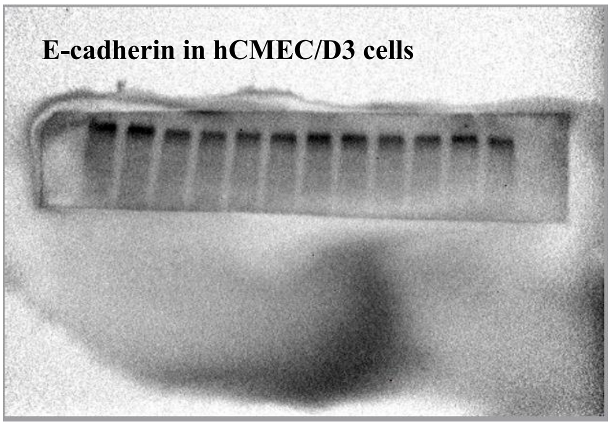
E-cadherin in hCMEC/D3 cells

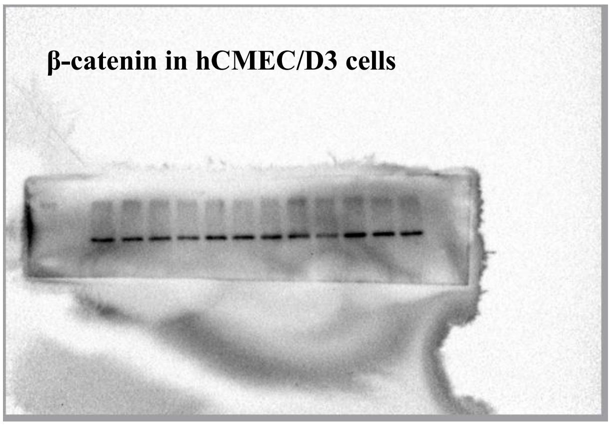
β-catenin in hCMEC/D3 cells

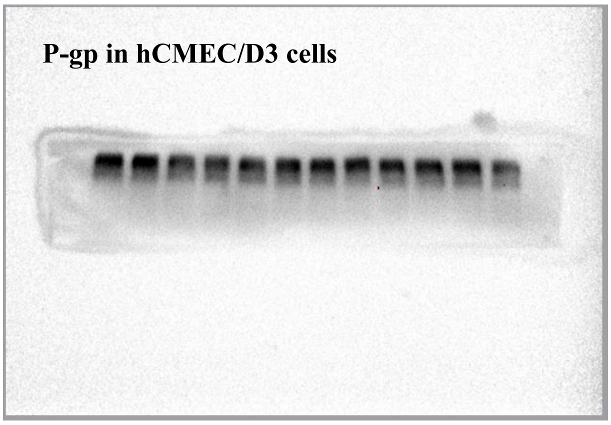
P-gp in hCMEC/D3 cells

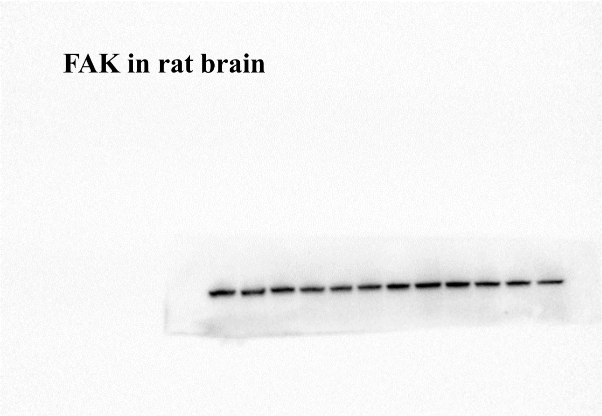
FAK in rat brain

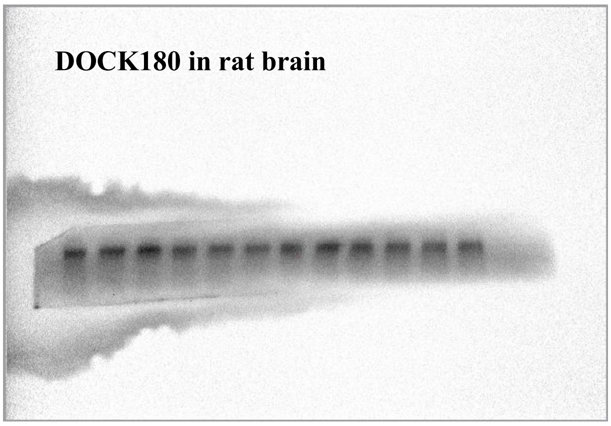
DOCK180 in rat brain

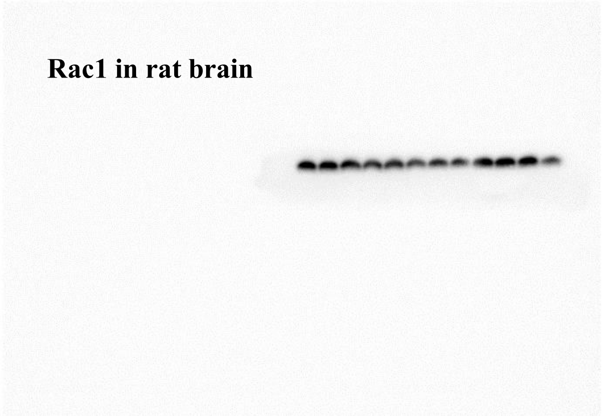
Rac1 in rat brain

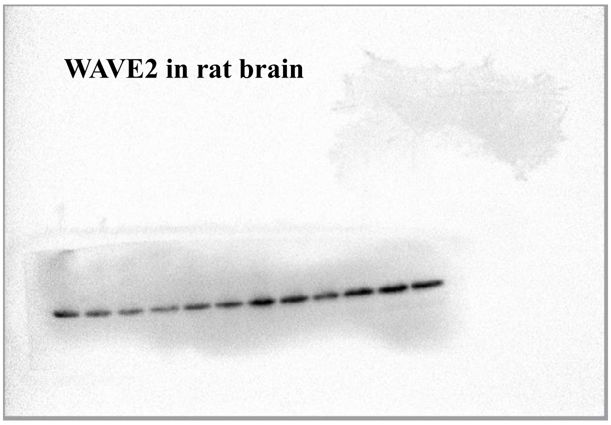
WAVE2 in rat brain

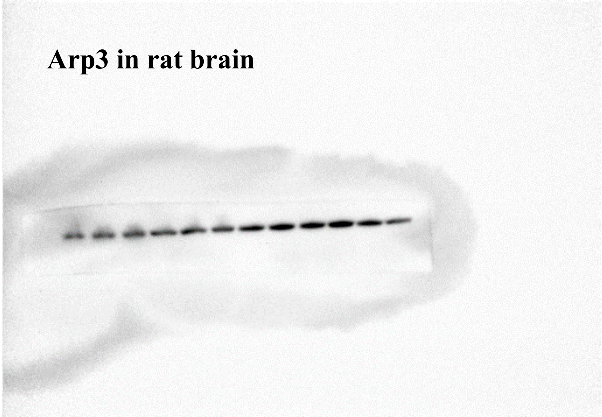
Arp3 in rat brain

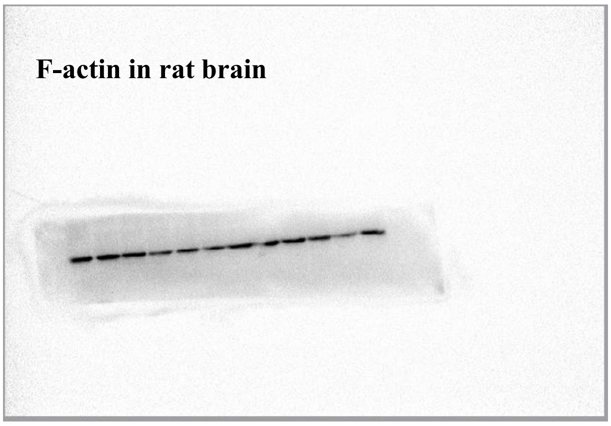
F-actin in rat brain

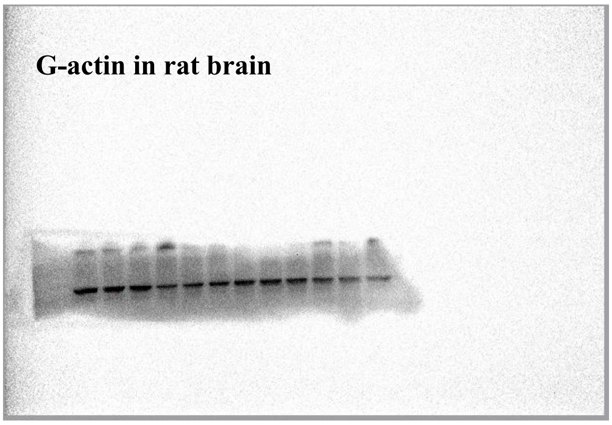
G-actin in rat brain

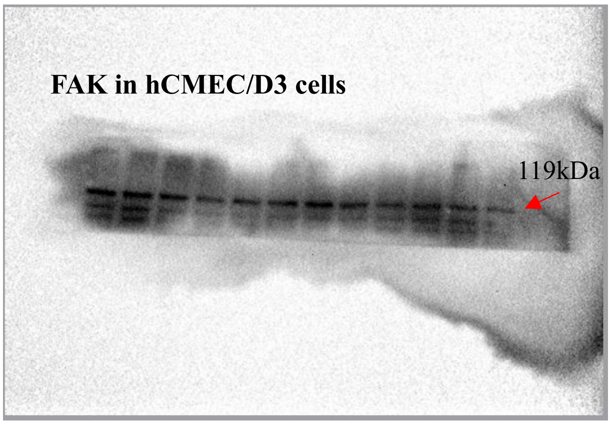
FAK in hCMEC/D3 cells
119kDa

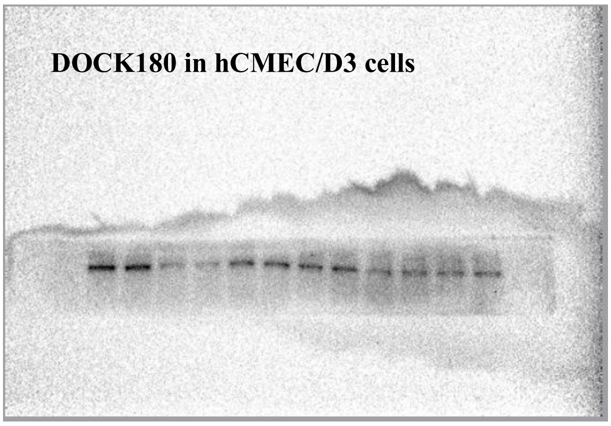
DOCK180 in hCMEC/D3 cells

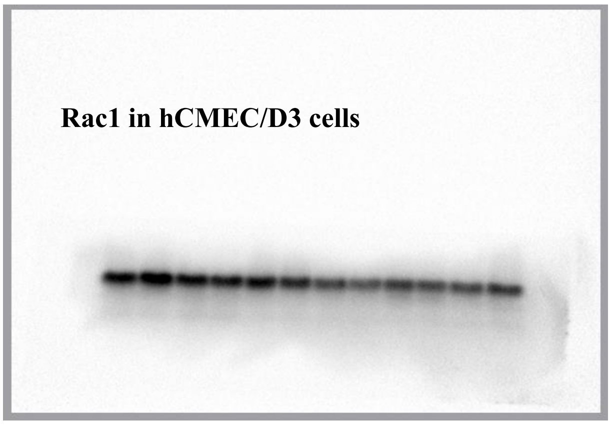
Rac1 in hCMEC/D3 cells

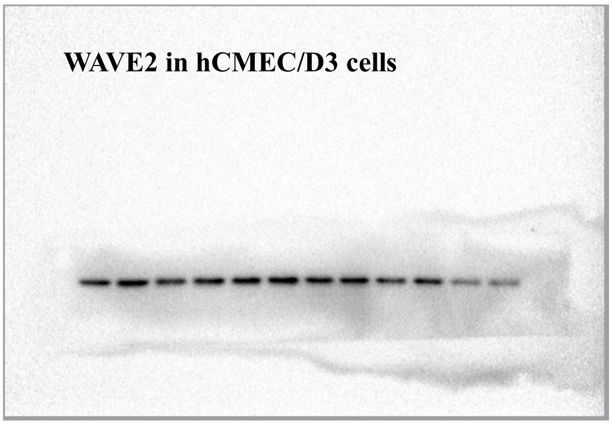
WAVE2 in hCMEC/D3 cells

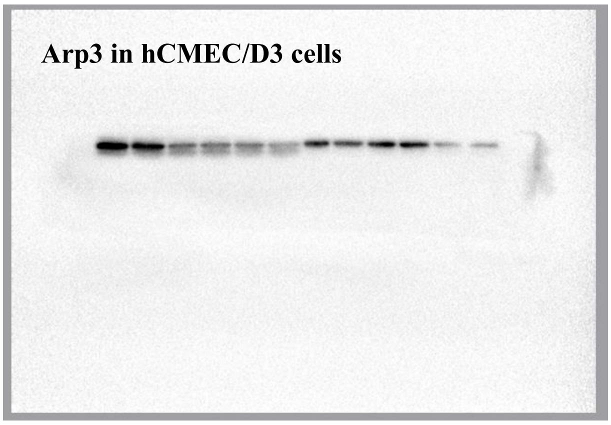
Arp3 in hCMEC/D3 cells

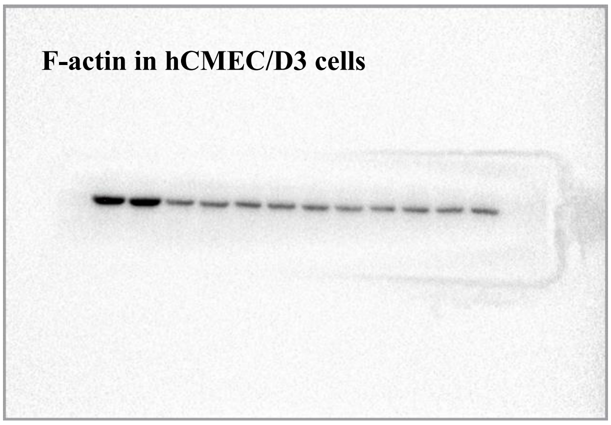
F-actin in hCMEC/D3 cells

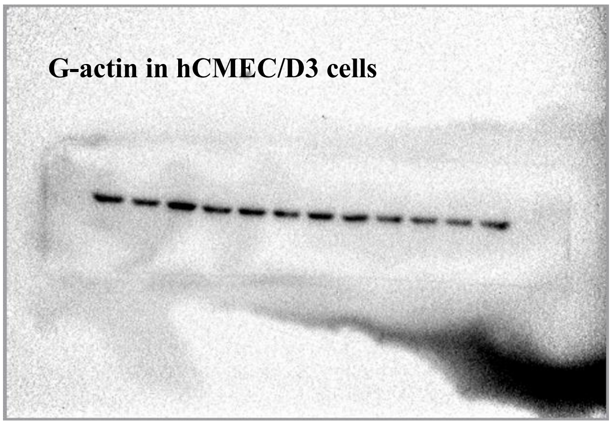
G-actin in hCMEC/D3 cells
